# Cell Cycle‐Specific Regulation of Centrosome Clustering Dynamics in Cancer Cells by the Multifunctional Kinesin HSET

**DOI:** 10.1002/advs.74651

**Published:** 2026-03-06

**Authors:** Po‐Pang Chen, Athira Saju, Chia‐Chou Wu, Tzu‐Han Weng, Su‐Yi Tsai, Tzu‐Lun Huang, Jia‐Ying Su, Chien‐Ling Lin, Yu‐Chun Lin, See‐Yeun Ting, Sheng‐hong Chen, Kuo‐Chiang Hsia

**Affiliations:** ^1^ Institute of Molecular Biology Academia Sinica Taipei Taiwan; ^2^ Institute of Biochemistry and Molecular Biology College of Life Sciences National Yang‐Ming Chiao‐Tung University Taipei Taiwan; ^3^ Molecular and Cell Biology Taiwan International Graduate Program and National Defense Medical Center Taiwan; ^4^ Department of Life Science National Taiwan University Taipei Taiwan; ^5^ Institute of Molecular Medicine National Tsing Hua University Hsinchu Taiwan

**Keywords:** centrosome, cancer, kinesin HSET, microtubule

## Abstract

Centrosome amplification in cancer cells produces excess centrosomes that cluster and decluster throughout the cell cycle, a process critical for tumorigenesis. Here, we identify the mitotic kinesin HSET as a multifaceted regulator of these dynamics in cancer cells. Beyond microtubule crosslinking, HSET co‐condensates with the centrosomal protein CDK5RAP2 and actively transports it toward microtubule minus ends. This directed delivery concentrates centrosomes at spindle poles, thereby limiting centrosome dispersal and contributing to centrosome coalescence. Notably, HSET‐driven transport enables effective transport of CDK5RAP2 condensates, independently of size, thereby overcoming cytosolic viscosity to cluster large foci of pericentriolar material within cells. Furthermore, HSET's ATP‐independent self‐assembly prevents centrosomal declustering, preserving centrosome integrity during mitotic progression. These activities position HSET as a critical regulator of centrosome clustering and integrity, with its mitosis‐specific upregulation providing another layer of cell cycle control over centrosome assembly and underscoring its potential as a target for cancer therapeutics.

## Graphical abstract

CDK5RAP2 associates with the kinesin HSET and forms co‐condensates. HSET motors drive the directional transport of CDK5RAP2 condensates toward microtubule minus ends. During mitosis, HSET self‐assembly further stabilizes clustered centrosomes, thereby enabling pseudo‐bipolar spindle formation in cancer cells with supernumerary centrosomes. Upon mitotic exit, APC‐dependent degradation and nuclear sequestration reduce cytoplasmic HSET levels, leading to centrosome dispersal during interphase.

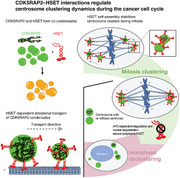

## 1 Introduction

The centrosome consists of a pair of cylindrical centrioles embedded in a protein‐rich matrix known as the pericentriolar material (PCM) [1]. Various microtubule‐nucleating factors, such as γ‐tubulin ring complexes (γ‐TuRCs), are deposited in the PCM [1, 2, 3, 4], allowing the centrosome to function as a microtubule‐organizing center (MTOC) and establish a mitotic bipolar spindle. Precise control of centrosome duplication, limited to once per cell cycle, is necessary to establish bipolarity of the spindle [5, 6]. This bipolarity ensures accurate chromosome segregation during mitosis, a fundamental process for genomic stability [7].

The presence of supernumerary centrosomes is a common feature of cancer cells [8, 9]. Such aberrations can arise through several mechanisms, including centrosome over‐duplication [8], centriole over‐elongation [10, 11], or PCM fragmentation [12]. Loss of centrosome structural integrity drives PCM fragmentation, giving rise to acentriolar centrosomes, which retain microtubule‐nucleating capacity [12]. The presence of multiple centrosomes can contribute to aneuploidy and chromosomal instability, which are critical drivers of intra‐tumor heterogeneity [13]. This heterogeneity is a hallmark of cancer, contributing to tumor recurrence and metastasis [14, 15].

Notably, supernumerary centrosomes challenge cell viability by promoting the formation of multipolar spindles during mitosis. To overcome this problem, cancer cells employ mechanisms to cluster extra centrosomes into two groups during mitosis, thereby forming a pseudo‐bipolar spindle. This process, known as centrosome coalescence, is critical for tumor initiation [8, 9]. Beyond mitosis, amplified centrosomes can cluster during interphase to facilitate cancer cell invasion [16]. Despite its role in tumor initiation and invasion, centrosome clustering must be reversed during the cell cycle to allow continued cancer cell proliferation [17, 18]. However, the regulatory mechanisms governing centrosome clustering during the cell cycle, which facilitate cancer initiation, progression, and invasion, and that contribute to intra‐tumor heterogeneity, remain poorly understood.

Additionally, as a membrane‐less organelle, the centrosome has also been proposed to act as a phase‐separated condensate that selectively concentrates biomolecules [19, 20, 21]. Many pericentriolar proteins, such as SPD‐5 (the *Caenorhabditis elegans* homolog of human CDK5 regulatory subunit associated protein 2, CDK5RAP2) [22], form phase‐separated liquid condensates, displaying properties of dynamic spontaneous self‐assembly. Hence, given their biophysical properties, including their reversible and dynamic nature [22, 23], centrosomes behave similarly to condensates. However, the mechanism by which centrosomes, with their micrometer‐scale diameters, are efficiently navigated to cluster within microtubule networks of nanometer‐sized mesh [24, 25] remains nuclear. Additionally, cytosolic viscosity, which increases exponentially with cargo size [26, 27, 28], limits the transport of larger cargos, prompting the question as to how such transport is coordinated with micrometer‐sized centrosomes within the constrained timeframe of mitosis.

Current models suggest that HSET, a minus end‐directed motor protein with limited processivity [29, 30, 31], facilitates centrosome coalescence in cancer cells by sliding and bundling microtubules during mitosis [32, 33, 34]. Additionally, formation of the PCM foci mediated by CDK5RAP2 mimics mitotic accumulation of the PCM matrix around centrioles [35], and assembly of acentriolar PCM is reliant on a conserved, microtubule‐dependent process [36, 37, 38]. Clustering of plant homologs of HSET has been proposed to facilitate retrograde transport in plants [39]. Moreover, CDK5RAP2, an evolutionarily conserved pericentriolar protein that localizes to the outer PCM [40, 41, 42, 43], interacts with HSET and thereby facilitates maintenance of mitotic spindle integrity [44]. Thus, we hypothesize that HSET‐dependent molecular mechanisms are responsible for centrosome clustering and that they regulate the structural integrity of clustered centrosomes during the mammalian cell cycle.

Here, our analysis of data reveals that HSET is expressed at high levels in various cancer types, and it is negatively correlated with cancer prognoses. We show that centrosome number varies in liver and breast cancer cell lines at all cell‐cycle stages, reflecting cycles of amplification and clustering that generate heterogeneity. Depletion of HSET or pharmacological inhibition of its activity during the G2/M cell cycle checkpoint not only promoted multipolar spindle formation, but also caused the centrosomes at spindle poles to become dispersed. These findings indicate that HSET actively transports and clusters centrosomes at spindle poles. We further show that CDK5RAP2 condensates cluster HSET, thereby enhancing its processivity. In turn, HSET transports CDK5RAP2 condensates toward the minus ends of microtubules, driving coalescence of CDK5RAP2‐containing PCM foci in cells. Beyond this cargo transport activity, HSET also exhibits properties of self‐assembly that stabilize the structural integrity of clustered centrosomes. Overall, our findings reveal that HSET's transport and stabilizing activities are critical to centrosome clustering and maintenance of their structural integrity, thereby contributing to tumor initiation, proliferation, and invasion, all of which are associated with poor patient outcomes.

## 2 Results

### 2.1 Centrosomes Undergo Clustering‐Declustering Transitions Throughout the Cell Cycle in Cancer Cells

**FIGURE 1:**
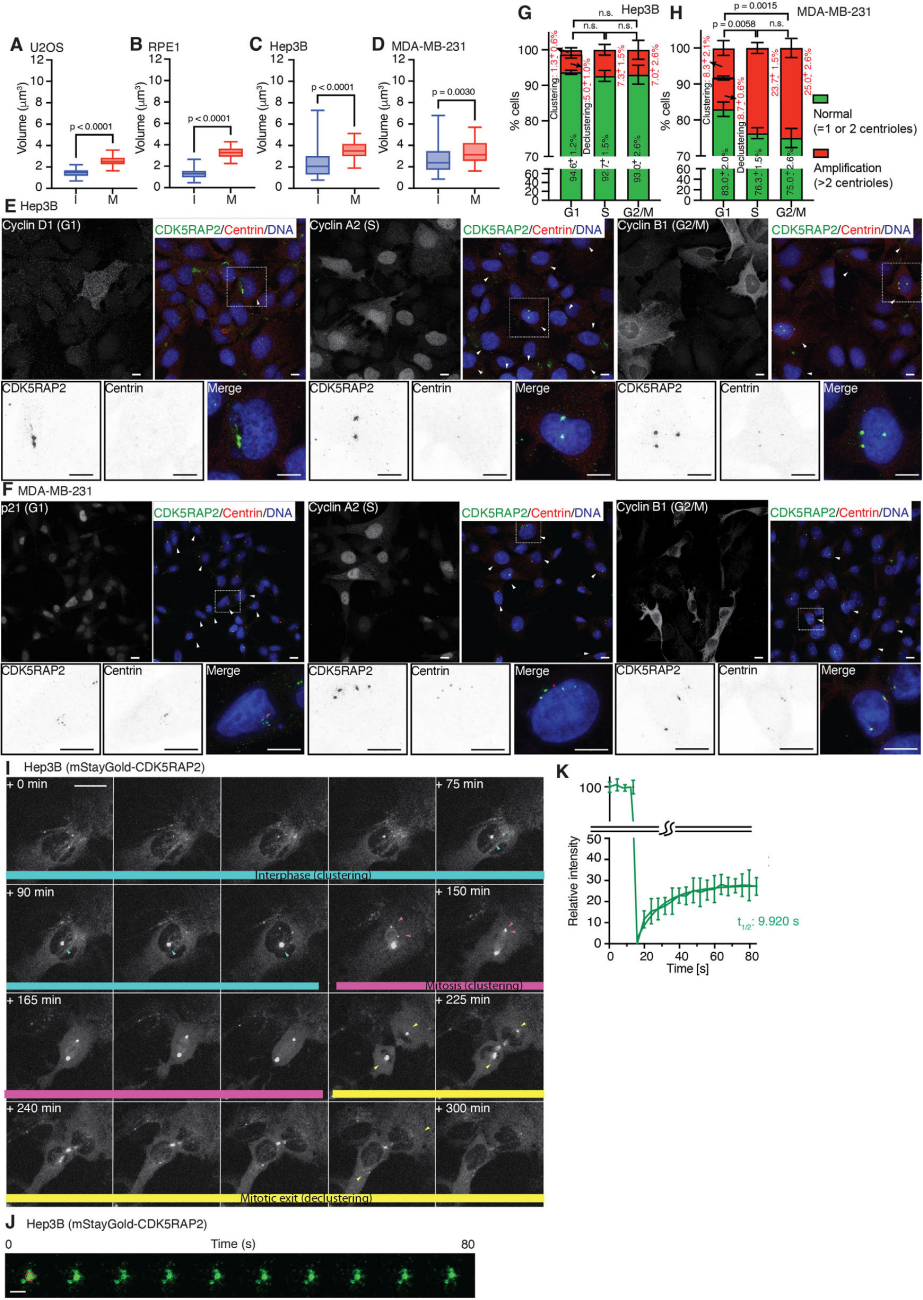
Centrosome heterogenicity in cancer cells. A–D) Quantification of centrosome volume using CDK5RAP2 signals during interphase (I) and mitosis (M) for U2OS (A), RPE1 (B), Hep3B (C), and MDA‐MB‐231 (D) cells, as shown in Figure S1A–D. Statistical significance was determined by two‐tailed Student's *t*‐test, with *p*‐values indicated. Detailed statistical results are provided in Table S5. E,F) Representative confocal immunofluorescence staining images of Hep3B (E) and MDA‐MB‐231 (F) cells, stained for cell‐cycle markers (Cyclin D1 and p21 for G1, Cyclin A2 for S, Cyclin B1 for G2/M) and CDK5RAP2 (green)/centrin (red)/DNA (blue). Cells positive for cell cycle markers (left panels) are indicated by arrows in the corresponding merged images (right panels). A representative cell with multiple centrosomes is highlighted in the boxed region and shown at higher magnification (inset). Scale bar: 10 µm. G,H) Percentages of Hep3B (G) and MDA‐MB‐231 (H) cells in each cell cycle stage with normal (1–2 centrioles) versus amplified (>2 centrioles) centrosomes are quantified, as determined from the representative images in (E) and (F). *n* = 300 cells for both lines; percentage values are indicated. The percentages of G1‐phase cells containing more than two centrioles with either clustered or declustered centrosomes are also shown. Statistical significance was determined by two‐tailed Student's *t*‐test, with *p*‐values indicated. n.s. indicates no significant difference. I) Time‐lapse confocal imaging of Hep3B cells expressing mStayGold‐CDK5RAP2, recorded from late G2 through mitotic exit. Centrosomes undergoing clustering and declustering at cell cycle stages are indicated. Blue and pick arrows mark centrosomes undergoing clustering, whereas yellow arrows indicate the centrosome disassembly. The numbers in the upper right corners indicate the elapsed time since initiation of time‐lapse imaging. Scale bar: 50 µm. J) mStayGold‐CDK5RAP2‐containing centrosomes in Hep3B cells were photo‐bleached and imaged. Photo‐bleach site is indicated by red circle. Scale bar: 2 µm. K) Quantitative analysis of mStayGold–CDK5RAP2 fluorescence recovery after photobleaching at centrosomes (*n* = 10). The half‐time of recovery (*t*1/2) was 9.92 s.

Centrosomes expand during the interphase‐to‐mitosis transition to facilitate spindle assembly [5, 6]. Accordingly, we found that mitotic centrosomes were typically larger than those of interphase centrosomes in U2OS and RPE1 cell lines, and the centrosomes within each stage displayed a relatively uniform size (Figure 1A,B; Figure S1A,B). In contrast, in cell lines reported to undergo centrosome amplification (i.e., Hep3B [45, 46] and MDA‐MB‐231 [11, 47]), the centrosomes in both interphase and mitosis exhibited substantial size heterogeneity (Figure 1C,D; Figure S1C,D). Notably, we observed an additional population of interphase centrosomes that were larger than their mitotic counterparts (Figure 1C,D; Figure S1C,D), indicating that centrosome clustering not only occurs during mitosis in cancer cells, but also in interphase. Furthermore, we conducted co‐immunofluorescence staining for the centrosome markers CDK5RAP2 and centrin, together with the cell cycle‐specific markers cyclin D1/p21(G1 phase), cyclin A2 (S phase), and cyclin B1 (G2/M phase). Both the Hep3B and MDA‐MB‐231 cells exhibited multiple centrosomes throughout all stages of the cell cycle (Figure 1E,F). Quantitative analysis revealed that a substantial proportion of the cells in each cell cycle phase contained more than two centrioles (Figure 1G,H). These results indicate that in addition to canonical centrosome duplication during S phase, a subset of extra centrosomes arises from declustering after mitotic exit (e.g., in G1 phase; ∼5% in Hep3B and ∼8.7% in MDA‐MB‐231). Moreover, among G1‐phase cells, a subset of single centrosomes contained multiple centrioles (Figure 1G,H; ∼1.3% in Hep3B and ∼8.3% in MDA‐MB‐231), indicating the presence of clustered centrosomes during interphase. Although the majority of CDK5RAP2 foci we analyzed were positive for centrin, we also observed a small population of CDK5RAP2‐positive foci lacking detectable centrioles (Figure S1E,F). These findings support the coexistence of distinct centrosome populations with or without centrioles in these cancer cell lines. These populations are likely generated by different mechanisms, such as via PCM fragmentation.

Next, we established stable Hep3B cell lines expressing mStayGold‐labeled CDK5RAP2 under the control of the chicken β‐actin promoter. mStayGold is an exceptionally bright and photostable fluorescent protein [48]. Thus, although the expression level of mStayGold‐tagged CDK5RAP2 was approximately one‐third of that of the endogenous protein (Figure S2A,B), fluorescence signals localized to CDK5RAP2 foci marked by centrin and PCNT (Figure S2C), enabling reliable long‐term live‐cell imaging. Live‐cell imaging revealed that multiple CDK5RAP2 foci of varying sizes clustered into fewer spots during interphase, prior to M phase (Figure 1I; Figure S2D; Video S1). These CDK5RAP2 foci further clustered during mitosis (Figure 1I; pink arrows). Notably, they underwent disassembly following cell division (Figure 1I; Figure S2D; Video S1). Taken together, these findings suggest that centrosome size and number fluctuate dynamically across all cell cycle stages, indicating that centrosomes in cancer cells undergo continuous cycles of clustering and declustering.

Next, we examined CDK5RAP2 dynamics in cancer cells using fluorescence recovery after photo‐bleaching (FRAP). Stable Hep3B cell lines exhibited slow recovery, reaching only ∼30% of the original fluorescence intensity after photo‐bleaching (Figure 1J,K; Figure S2E). The recovery half‐time was ∼10 seconds, with an immobile fraction of 70% (Figure 1K). To assess the dynamics of CDK5RAP2 in non‐cancer cells, we also generated stable RPE1 cell lines expressing mStayGold‐labeled CDK5RAP2. Consistent with *C. elegans* SPD‐5, a functional homolog of human CDK5RAP2 [22], CDK5RAP2 in non‐cancer cells exhibited low turnover and limited recovery at centrosomes (Figure S2F–H). These results indicate that the CDK5RAP2 in both the cancer and non‐cancer cells displays limited dynamism.

### 2.2 HSET‐Dependent Centrosome Clustering is Critical for Bipolar Spindle Formation and Centrosome Integrity

**FIGURE 2:**
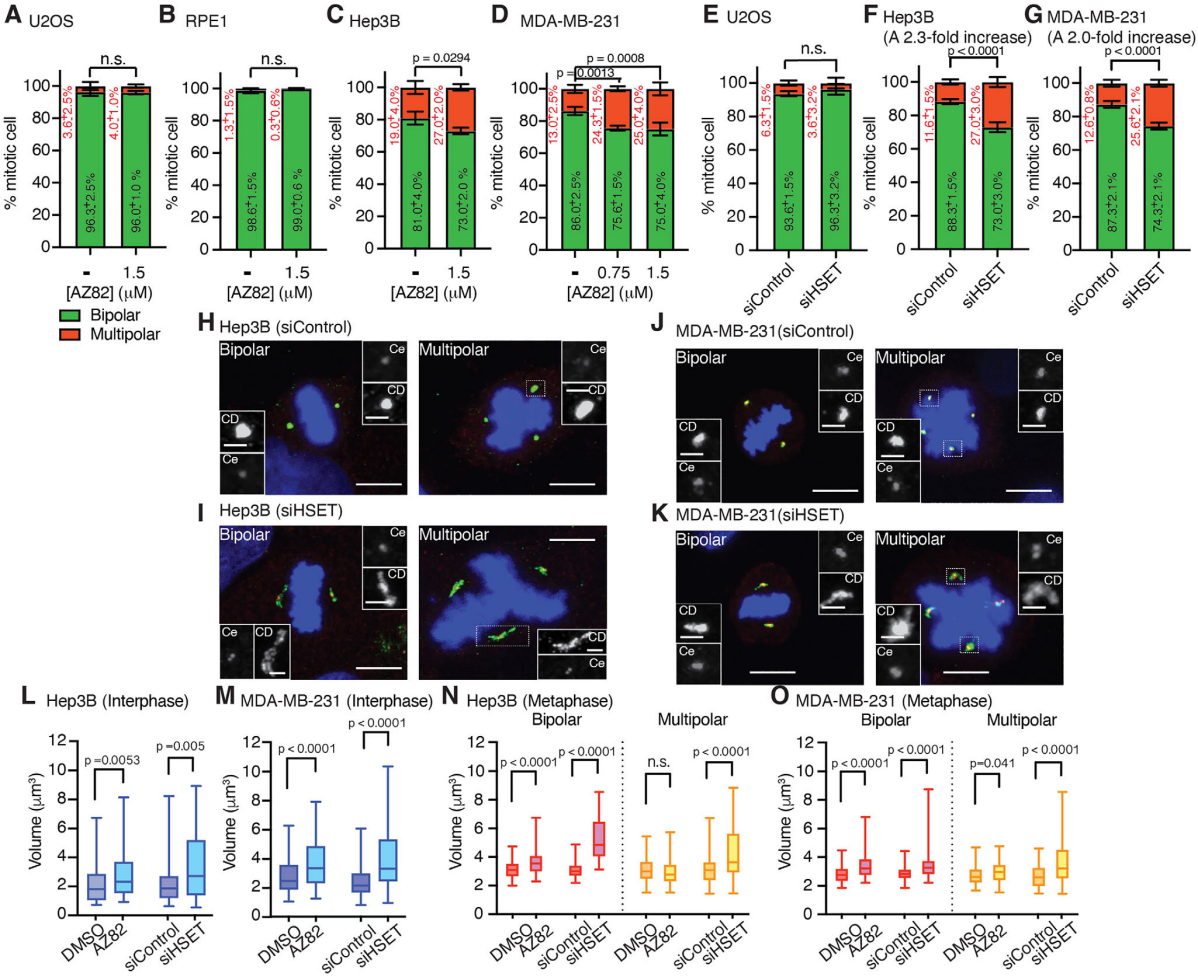
HSET modulates bipolar spindle formation and centrosome integrity. A–D) Percentages of bipolar and multipolar spindles in mitotic cells for U2OS (A), RPE1 (B), Hep3B (C), and MDA‐MB‐231 (D) cells treated with the indicated concentrations of AZ82. *n* = 300 cells for all cell lines; percentage values are indicated. Statistical significance was determined by two‐tailed Student's *t*‐test, with *p*‐values indicated. n.s. indicates no significant difference. E‐G) Percentages of bipolar and multipolar spindles in mitotic U2OS (E), Hep3B (F), and MDA‐MB‐231 (G) cells following siRNA‐mediated HSET depletion. *n* = 300 cells for all cell lines; percentage values are indicated. Statistical significance was determined by two‐tailed Student's *t*‐test, with *p*‐values indicated. n.s. indicates no significant difference. H–K) Representative immunofluorescence confocal images of bipolar and multipolar spindles in Hep3B (H,I) and MDA‐MB‐231 (J,K) cells transfected with siControl or siRNA targeting HSET. Centrioles were stained by centrin (Ce; red), the pericentriolar material by CDK5RAP2 (CD; green), and DNA by DAPI (blue). Merged images are shown, with centrosomes at spindle poles highlighted at higher magnification (inset). Scale bar: 10 µm; 2 µm (insets). L,M) Quantification of CDK5RAP2‐positive centrosome volume in interphase Hep3B (L) and MDA‐MB‐231 (M) cells under DMSO, AZ82, siControl, or siHSET treatment conditions. N,O) Quantification of CDK5RAP2‐positive centrosome volume in bipolar and multipolar spindles during metaphase for Hep3B (N) and MDA‐MB‐231 (O) cells under DMSO, AZ82, siControl, or siHSET treatment conditions. Statistical significance was determined by two‐tailed Student's *t*‐test, with *p*‐values indicated. n.s. indicates no significant difference. Detailed statistical results are provided in Table S5.

Next, we applied AZ82, an HSET inhibitor that blocks its ATPase activity [49, 50, 51], as well as performed siRNA knockdown to suppress HSET's function in centrosome clustering, in MDA‐MB‐231 and Hep3B cells. Quantification of mitotic cells revealed that the AZ82 treatment significantly increased the frequency of multipolar spindles in both Hep3B and MDA‐MB‐231 cells (1.4‐ and 1.8‐fold increases, respectively), but not in U2OS or RPE1 cells (Figure 2A–D; Figure S3A–D). Consistently, siRNA‐mediated HSET depletion also resulted in 2‐ and 2.3‐fold increases in the numbers of multipolar spindles in both cancer cell lines compared to siControl conditions (Figure 2E–K). Although the Hep3B and MDA‐MB‐231 cells exhibited higher HSET expression levels than RPE1 cells (Figure S3E,F), HSET expression could still be efficiently knocked down in both cancer cell lines, as confirmed by Western blot analysis (Figure S3G). Notably, neither siRNA knockdown nor AZ82‐mediated activity inhibition of HSET in either of the cancer cell lines changed overall CDK5RAP2 protein levels, as corroborated by Western blotting (Figure S3H–M).

Beyond the increase in spindle pole numbers, both the AZ82 and siRNA knockdown treatments caused CDK5RAP2 signals to become more dispersed during interphase and metaphase (Figure 2H–O). Notably, these splayed CDK5RAP2 signals were observed in both bipolar and multipolar spindles in both of the cancer cell lines we examined (Figure 2N,O), although no significant difference was detected among multipolar spindles in Hep3B cells. Previous studies have shown that dynein transports aggregated NuMA along spindle microtubules to spindle poles, as inhibiting dynein activity prevented NuMA accumulation at the poles [52, 53]. Accordingly, our findings lead us to propose that, apart from sliding and bundling microtubules during mitosis to facilitate centrosome clustering and establish bipolar spindles [32], HSET actively promotes the accumulation of centrosomal materials at the poles in cancer cells. Importantly, siRNA‐mediated knockdown of HSET resulted in a pronounced increase in centrosome volume in multipolar spindles in both cell lines compared to AZ82 treatment (Figure 2N,O). These differences indicate that HSET functions beyond its motor activity may contribute to centrosome clustering or the maintenance of centrosome integrity.

Despite AZ82 treatment or siRNA‐mediated HSET knockdown, the percentages of cells containing more than two centrioles during interphase or within bipolar and multipolar spindles remained comparable (Figure S3N–S). These results indicate that HSET does not regulate centriole number. Instead, they imply that the dispersed CDK5RAP2 signals observed in bipolar and multipolar spindles arise from amplified centrosomes that fail to cluster efficiently.

### 2.3 CDK5RAP2 Forms Condensates With HSET In Vitro

**FIGURE 3:**
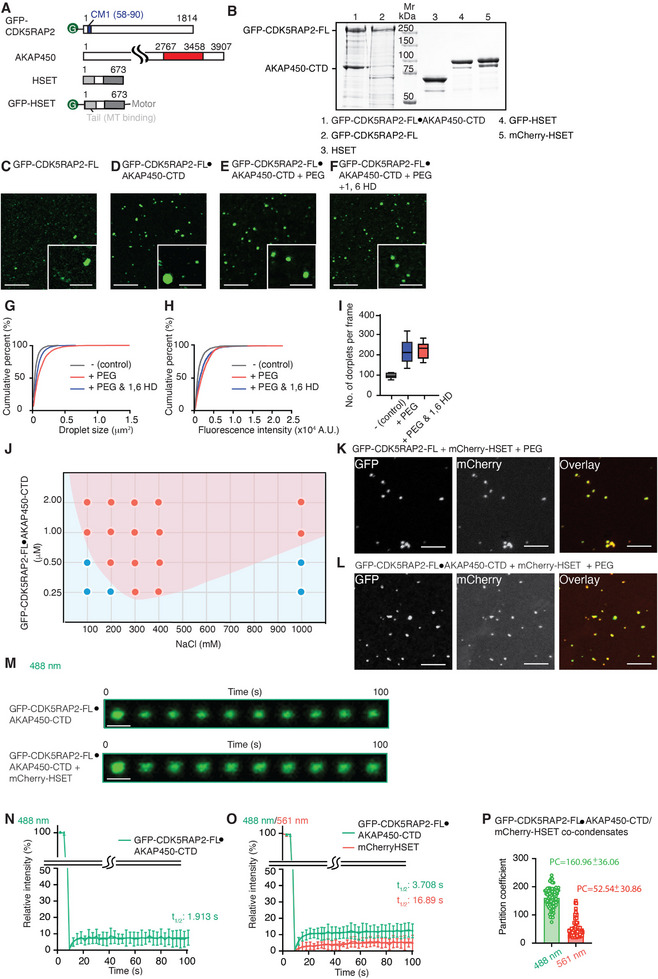
Formation of CDK5RAP2 and HSET co‐condensates in vitro. A) Schematic representations of the proteins used in TIRF‐based in vitro constitution experiments. The CM1 motif of CDK5RAP2 that interacts with γ‐TuRC is highlighted in blue. The region of AKAP450 co‐expressed with CDK5RAP2 is colored red. The microtubule‐binding and motor domains of HSET are depicted in light and dark gray, respectively. Residue numbers and GFP tags are indicated. B) Purified recombinant full‐length GFP‐CDK5RAP2 (referred to as GFP‐CDK5RAP2‐FL), the GFP‐CDK5RAP2•AKAP450 (2767‐3458) complex (referred to as GFP‐CDK5RAP2‐FL•AKAP450‐CTD), GFP‐HSET, and HSET were analyzed by SDS‐PAGE and stained with Coomassie blue. C) A representative TIRF image of GFP‐CDK5RAP2‐FL incubated in buffer without PEG and analyzed after 5 min. Scale bar: 5 µm. D,E) A representative TIRF image of GFP‐CDK5RAP2‐FL•AKAP450‐CTD incubated in buffer in the absence (D) or presence (E) of 4% PEG and analyzed after 5 min. Scale bar: 5 µm. F) A representative TIRF image of GFP‐CDK5RAP2‐FL•AKAP450‐CTD incubated in buffer in the presence of 4% PEG and 4% 1,6 HD. Scale bar: 5 µm. G–I) Quantification of droplet size (G), fluorescence signal intensity of droplets (H), and droplet density (I) for GFP‐CDK5RAP2‐FL•AKAP450‐CTD condensates under the indicated conditions. Control: n = 1026; +PEG: n = 1943; +PEG&1,6 HD n = 1963. J) Phase diagram of GFP‐CDK5RAP2‐FL•AKAP450‐CTD under varying protein and salt concentrations. Red and blue dots indicate conditions where condensate droplets were observed or absent, respectively. The line separating the two regions indicates the approximate phase boundary. K,L) Representative TIRF images of GFP‐CDK5RAP2‐FL (K) or GFP‐CDK5RAP2‐FL•AKAP450‐CTD (L) incubated in buffer containing 4% PEG in the presence of mCherry‐HSET. A merged fluorescence image is shown in the right panel. Scale bar: 5 µm. M) FRAP analysis of GFP–CDK5RAP2‐FL•AKAP450‐CTD condensates formed in the absence (top) or presence (bottom) of HSET. Scale bar: 1 µm. N,O) Recovery kinetics of GFP‐CDK5RAP2‐FL•AKAP450‐CTD (green) and mCherry‐HSET (orange) after bleaching of entire condensates, in the absence (N) or presence (O) of HSET (*n* = 10 for both conditions). Half‐times of fluorescence recovery (*t*1/2) are indicated. P) Partitioning coefficients of GFP‐CDK5RAP2‐FL and mCherry‐HSET in GFP‐CDK5RAP2‐FL•AKAP450‐CTD/mCherry co‐condensates (n = 78).

*C. elegans* SPD‐5 has been demonstrated previously to form selective condensates in vitro [22]. Additionally, clustering of HSET homologs in plants enhances its processivity, facilitating retrograde transport [39]. To biochemically characterize if CDK5RAP2 forms condensates and whether it can be transported by HSET, we applied an in vitro reconstitution system using recombinant proteins. First, we expressed and purified individual HSET and CDK5RAP2 proteins, with or without fluorescence tags, in insect cells (Figure 3A,B), but the yield and quality of GFP‐tagged full length CDK5RAP2 (hereafter GFP‐CDK5RAP2‐FL) were relatively low (Figure 3B).

AKAP450 is a centrosome‐ and Golgi‐localized protein, and its C‐terminus has been shown previously to interact with CDK5RAP2 [54]. Importantly, neither siRNA‐mediated knockdown nor AZ82‐mediated inhibition of HSET activity altered AKAP450 protein levels in either of the cancer cell lines, similar to the effects observed for CDK5RAP2 (Figure S3H–M). In addition, AKAP450 maintained its cellular co‐localization with CDK5RAP2 in MDA‐MB‐231 cells under both treatment conditions (Figure S3T–W). Given that AKAP450 has been reported previously to enhance the cellular stability of CDK5RAP2 [55], we co‐expressed CDK5RAP2 together with AKAP450 to improve protein yield and quality. This strategy produced a soluble and stable hetero‐dimer of GFP‐tagged full‐length CDK5RAP2 linked to the AKAP450 C‐terminal (amino acids 2767–3458), which we refer to hereafter as GFP‐CDK5RAP2‐FL•AKAP450‐CTD (Figure 3A,B). Western blot analyses and mass spectrometry confirmed the presence of both proteins (Figure S4A–C).

We observed that both GFP‐CDK5RAP2‐FL and GFP‐CDK5RAP2‐FL•AKAP450‐CTD assembled into micron‐scale condensates (Figure 3C,D). Since GFP‐CDK5RAP2‐FL•AKAP450‐CTD exhibited better protein quality and AKAP450‐CTD alone did not form condensates (Figure S4D), we used GFP‐CDK5RAP2‐FL•AKAP450‐CTD for subsequent condensate‐related experiments. Addition of polyethylene glycol 3350 (PEG3350) (4%) increased the fluorescence signal intensity, as well as the number and density of condensate puncta (Figure 3E,G–I). Notably, treatment with 1,6‐hexanediol, which disrupts hydrophobicity‐dependent condensates [56], only minimally reduced CDK5RAP2‐FL•AKAP450‐CTD condensate formation (Figure 3F–I). Moreover, phase diagram analyses revealed that GFP‐CDK5RAP2‐FL•AKAP450‐CTD condensate formation was dependent on salt concentration (Figure 3J; Figure S4E), indicating that electrostatic interactions contribute significantly to condensate assembly.

Furthermore, mCherry‐labeled HSET formed co‐condensates with both GFP‐CDK5RAP2‐FL and GFP‐CDK5RAP2‐FL•AKAP450‐CTD in the presence of PEG (Figure 3K,L), corroborating their direct interaction [44]. FRAP analysis showed that GFP‐CDK5RAP2‐FL•AKAP450‐CTD in condensates, with or without HSET, only partially recovered after bleaching (Figure 3M–O), consistent with the reversible but low dynamic character of CDK5RAP2 observed in cancer cells (Figure 1J,K). An analysis of partition coefficients further revealed preferential partitioning and relative co‐enrichment of GFP‐ and mCherry‐tagged proteins within the condensates (Figure 3P), supporting co‐partitioning and co‐existence of HSET and CDK5RAP2 within these assemblies.

### 2.4 The N‐Terminal Coiled‐Coil Region of CDK5RAP2 Mediates Condensate Formation

To identify the region of CDK5RAP2 responsible for condensate formation, we focused on the N‐terminal half of the protein, as the C‐terminus of CDK5RAP2 is known to be involved in centrosomal and Golgi localization [57]. We generated multiple GFP‐fused CDK5RAP2 N‐terminal fragments (Figure S5A). Among them, GFP‐CDK5RAP2 (248‐530) and GFP‐CDK5RAP2 (1‐530) robustly formed condensates (Figure S5B–G), characterized by greater fluorescence signal intensity, larger droplet size, and increased puncta density in the presence of PEG (Figure S5H–J). Phase diagram and FRAP analyses further confirmed that GFP‐CDK5RAP2 (1‐530) condensate formation is salt‐dependent (Figure S5K,L), and that GFP‐CDK5RAP2 (1‐530) and mCherry‐HSET exhibited reversible but low dynamic behavior, similar to GFP‐CDK5RAP2‐FL•AKAP450‐CTD (Figure S5M–P). Biochemical characterization showed that CDK5RAP2 (1‐530) displayed a sharp mono‐dispersed peak in size exclusion chromatography (SEC; Figure S5Q), with analytic ultracentrifugation with sedimentation velocity (SV‐AUC) revealing a molecular mass of ∼92 kDa (Figure S5R). Protein secondary structure prediction by PSIPRED [58, 59] and superimposition of five structural models predicted by AlphaFold3 [60] indicated that the region of CDK5RAP2 from residues 248 to 530 predominantly comprises α‐helices (Figure S5S,T). These findings indicate that the N‐terminal coiled‐coil region (residues 248–530) of CDK5RAP2 facilitates condensate formation. Therefore, CDK5RAP2 condensate formation is mediated by electrostatic interactions within the long N‐terminal coiled‐coil dimerization region.

### 2.5 HSET Actively Transports GFP‐CDK5RAP2‐FL•AKAP450‐CTD Condensates to the Minus Ends of Microtubules

**FIGURE 4:**
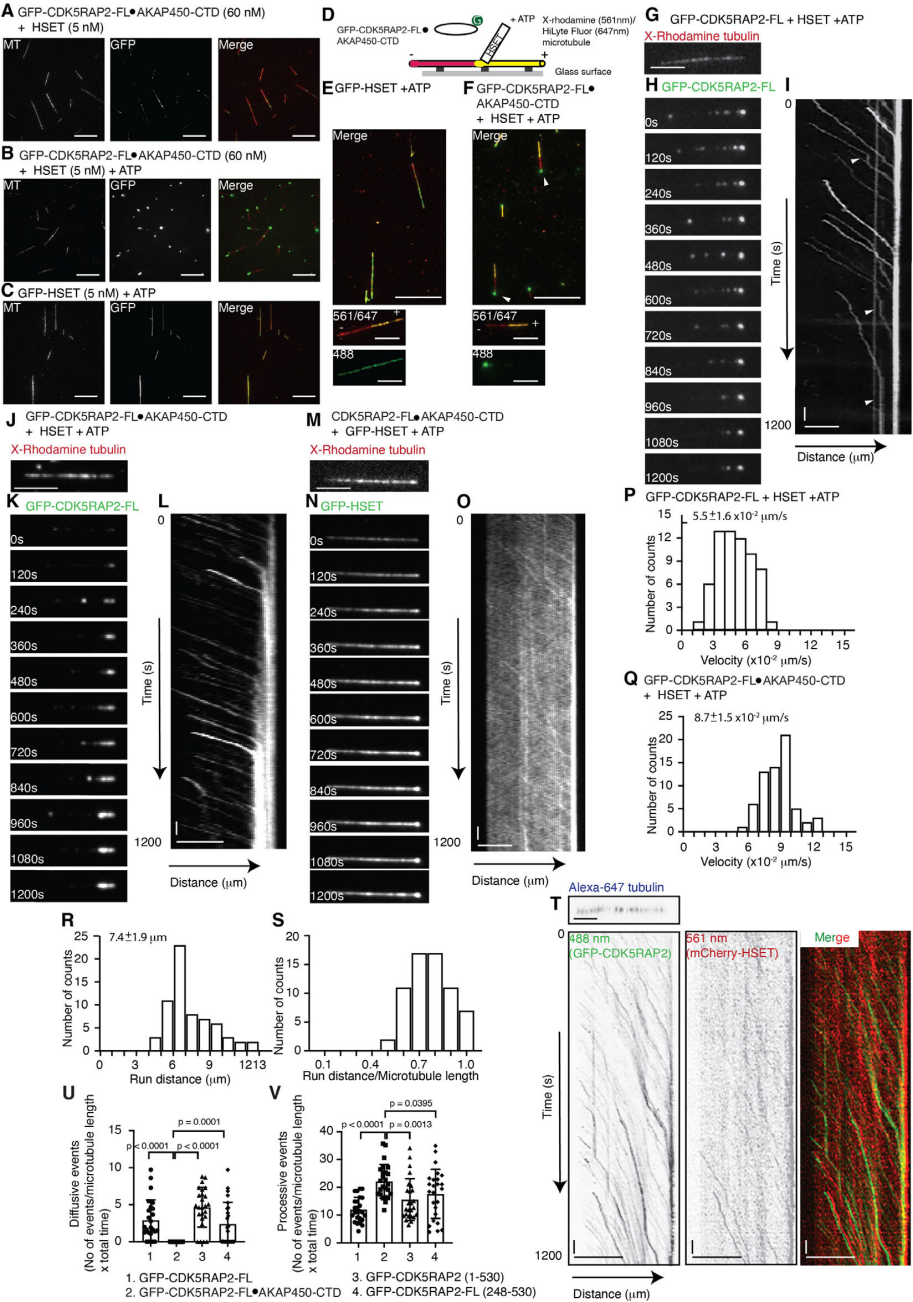
HSET actively transports CDK5RAP2 condensates. A,B) Representative TIRF images of GMPCPP‐stabilized microtubules (X‐rhodamine‐ and biotin‐labeled) immobilized on a glass surface incubated with GFP‐CDK5RAP2‐FL•AKAP450‐CTD in the presence of HSET (A) without or (B) with ATP. Scale bar: 10 µm. C) Representative TIRF images of GMPCPP‐stabilized microtubules (X‐rhodamine‐ and biotin‐labeled) immobilized on a glass surface incubated with GFP‐HSET and ATP. Scale bar: 10 µm. D) Schematic summary of GFP‐CDK5RAP2‐FL•AKAP450‐CTD recruitment to the polarity‐marked microtubules. Polarity‐marked microtubules were immobilized on a glass surface, incubated with GFP‐CDK5RAP2‐FL•AKAP450‐CTD (green dot) in the presence of HSET, and imaged using TIRF microscopy. First‐color microtubules are depicted in red, and second‐color microtubules are shown in yellow. Red and yellow represent X‐rhodamine 561 and HiLyte Fluor 647, respectively. Polarity is indicated by “+” or “−”. E) Representative TIRF images of polarity‐marked microtubules incubated with GFP‐HSET in the presence of ATP, shown in the indicated fluorescence channels. Scale bar: 10 µm. F) Representative TIRF images of polarity‐marked microtubules incubated with GFP‐CDK5RAP2‐FL•AKAP450‐CTD in the presence of HSET and ATP. Arrows highlight the accumulation of GFP signals at the microtubule tip. Scale bar: 10 µm. G,H) Representative time‐lapse TIRF images of a microtubule (G) and associated GFP‐CDK5RAP2‐FL (H) in the presence of HSET during end accumulation with ATP. Scale bars: 5 µm. I) Kymograph corresponding to the time‐lapse sequence in (H). J,K) Representative time‐lapse TIRF images of a microtubule (J) and associated GFP‐CDK5RAP2‐FL•AKAP450‐CTD (K) in the presence of HSET during end accumulation with ATP. Scale bars: 5 µm. L) Kymograph corresponding to the time‐lapse sequence in (K). M,N) Representative time‐lapse TIRF images of a microtubule (M) and associated GFP‐HSET (N) in the presence of CDK5RAP2‐FL•AKAP450‐CTD during end accumulation with ATP. Scale bars: 5 µm. O) Kymograph corresponding to the time‐lapse sequence in (N). P) Histograms of velocity for GFP‐CDK5RAP2‐FL in the presence of HSET. Mean and standard deviation were determined from data pooled from three independent experiments (66 counts per measurement). Differences were assessed statistically by two‐tailed Student's *t*‐test, with *p*‐values indicated. Q‐S) Histograms of velocity (Q), run distance (R), and the ratio of run distance over microtubule length (S) for GFP‐CDK5RAP2‐FL•AKAP450‐CTD in the presence HSET. Mean and standard deviation were determined from data pooled from three independent experiments (65 counts per measurement). Differences were assessed statistically by two‐tailed Student's *t*‐test, with *p*‐values indicated. T) Representative TIRF images of a microtubule corresponding to the kymograph analysis of GFP‐CDK5RAP2‐FL•AKAP450‐CTD and mCherry‐HSET in the presence of ATP. Kymographs from the 488 nm channel (left), 561 nm channel (middle), and the merged channels (right) are shown. Scale bars: 5 µm. U,V) Quantification of diffusive (U) and processive (V) events for the indicated constructs. Events were normalized to microtubule length and total imaging time. The data represent a pooled analysis from three independent experiments (n = 25). Mean values and standard deviations were analyzed statistically using a two‐tailed Student's *t*‐test, with the corresponding *p*‐values indicated.

We next employed TIRF microscopy to acquire snapshot images of the distribution of GFP‐CDK5RAP2‐FL•AKAP450‐CTD on single microtubules in the presence of HSET. GFP‐CDK5RAP2‐FL•AKAP450‐CTD (60 nm) alone presented weak fluorescence signals that were distributed along GMPCPP‐stabilized microtubules that had been immobilized on coverslips (Figure S6A). Incubating GFP‐CDK5RAP2‐FL•AKAP450‐CTD with HSET and without ATP supplementation increased GFP fluorescence signal intensity on microtubules in a HSET concentration‐dependent manner (Figure 4A; Figure S6A–C). These results support two key points: 1) the binding sites of GFP‐CDK5RAP2‐FL•AKAP450‐CTD and HSET on microtubules do not overlap; and 2) HSET enhances the duration of GFP‐CDK5RAP2‐FL•AKAP450‐CTD on microtubules. Remarkably, when both GFP‐CDK5RAP2‐FL•AKAP450‐CTD and HSET were incubated with single microtubule filaments in the presence of ATP, we observed micron‐sized accumulations at the microtubule ends, as demonstrated by line scans (Figure 4B; Figure S6D,E). GFP‐HSET alone displayed no preference for microtubule binding in the presence of ATP, as observed through line scans (Figure 4C; Figure S6F). Thus, ATP‐dependent HSET activity is essential for targeting GFP‐CDK5RAP2‐FL•AKAP450‐CTD to microtubules and enabling it to form micron‐scale accumulations at their tips.

We visualized the localization of the fluorescent GFP‐CDK5RAP2‐FL•AKAP450‐CTD on polarity‐marked microtubules in the presence of HSET (Figure 4D). GFP‐HSET uniformly bound along microtubule filaments in the presence of ATP (Figure 4E). However, under identical experimental conditions, the fluorescent GFP‐CDK5RAP2‐FL•AKAP450‐CTD specifically accumulated at the microtubule minus ends marked by X‐rhodamine (indicated by arrows in Figure 4F), indicating that HSET facilitates the localization of fluorescent GFP‐CDK5RAP2‐FL•AKAP450‐CTD at microtubule minus ends.

To determine if GFP‐CDK5RAP2‐FL•AKAP450‐CTD forms condensates and can be actively transported under microtubule‐dependent transport assay conditions, GFP‐CDK5RAP2‐FL•AKAP450‐CTD was examined in a buffer containing 1x BRB80. Consistent with the observations presented in Figure 3K–N, and regardless of the presence or absence of HSET, GFP‐CDK5RAP2‐FL•AKAP450‐CTD also assembled into micron‐scale condensates displaying low intrinsic dynamism (Figure S6H–K). Next, we conducted fluorescence time‐lapse imaging to assess condensate dynamics. The resulting time‐lapse sequences of GFP‐CDK5RAP2‐FL or GFP‐CDK5RAP2‐FL•AKAP450‐CTD in the presence of HSET demonstrated that the process of accumulation was initiated at the microtubule tip and expanded toward the opposite end of the filament (Figure 4G,H,J,K; Video S2 (for GFP‐CDK5RAP2‐FL•AKAP450‐CTD)). Kymographs illustrated continuous transportation and fusion of GFP‐labeled condensates with different fluorescent intensities at microtubule tips, resulting in increased puncta accumulation (Figure 4I,L; Figure S6L–Q). Quantification of condensate movement showed average velocities of 5.5 ± 1.6 × 10^−2^ µm/s for GFP‐CDK5RAP2‐FL and 8.7 ± 1.5 × 10^−2^ µm/s for GFP‐CDK5RAP2‐FL•AKAP450‐CTD (Figure 4P,Q). Note that, in the presence of AZ82, kymograph analyses revealed that the GFP‐CDK5RAP2‐FL•AKAP450‐CTD condensates remained associated with microtubules but were immobile (Figure S6R,S). These results indicate that although the condensates can still bind to microtubules, HSET‐dependent transport activity is inhibited by AZ82.

Under the same conditions, time‐lapse sequences of CDK5RAP2‐FL•AKAP450‐CTD and GFP‐HSET also showed GFP fluorescence signal accumulating at the microtubule tips (Figure 4M,N; Figure S6G). Despite a significant proportion of GFP‐HSET binding to microtubules without end accumulation, we did observe continuous streaming of GFP signals along microtubules toward their ends, with a velocity comparable to that determined for GFP‐CDK5RAP2 movement (Figure 4O; Figure S6T). The average travel distance of the GFP‐CDK5RAP2‐FL•AKAP450‐CTD condensates was measured at 7.4 ± 1.9 µm, covering a substantial proportion of the microtubule length used in our experiments (Figure 4R,S). Notably, the velocity of HSET‐mediated transport did not diminish in accordance with increasing GFP‐CDK5RAP2‐FL•AKAP450‐CTD condensate size (Figure S6U), enabling this transport mechanism to deliver large cargos (e.g., centrosomes) efficiently (also see the Discussion).

Next, we applied three‐color TIRF imaging using GFP‐CDK5RAP2‐FL•AKAP450‐CTD, mCherry‐HSET, and HiLyte‐647‐labeled microtubules. Kymograph analysis of time‐lapse sequences revealed coaligned diagonal trajectories of GFP and mCherry fluorescence signals (Figure 4T), corroborating that GFP‐CDK5RAP2‐FL•AKAP450‐CTD and HSET co‐migrate along microtubules toward the tips. Notably, under identical acquisition conditions, mCherry‐HSET alone did not exhibit diagonal trajectories toward the microtubule ends (Figure S6X,Y). However, when imaged using shorter acquisition intervals, short, non‐directional processive events were detectable (Figure S6V,W). Taken together, these findings indicate that end accumulation occurs through the directional transport of CDK5RAP2 condensates to microtubule minus ends by HSET.

### 2.6 The N‐Terminus of CDK5RAP2 Mediates HSET‐Dependent Transport

Previous pull‐down experiments have shown that CDK5RAP2 residues 300–700 interact with HSET [44]. Since we have already shown that GFP‐CDK5RAP2 (248‐530) and GFP‐CDK5RAP2 (1‐530) form condensates (Figure S5C,D), we conducted TIRF‐based assays to establish if droplets formed by these constructs could also be trafficked to microtubule ends. Similar to CDK5RAP2‐FL‐hosting condensates, both GFP‐CDK5RAP2 (248‐530) and GFP‐CDK5RAP2 (1‐530) accumulated at microtubule ends in the presence of HSET and ATP (Figure S7A–D). Moreover, both truncation constructs were delivered by HSET with velocities and run distances comparable to the CDK5RAP2‐FL‐hosting condensates (Figure S7E,F). Furthermore, kymographs showed that GFP‐CDK5RAP2‐FL and the two truncation constructs frequently exhibited diffusion events (arrows in Figure 4I,U; Figure S7B,D), which were rarely observed for GFP‐CDK5RAP2‐FL•AKAP450‐CTD (Figure 4L). Among these four tested constructs, GFP‐CDK5RAP2‐FL•AKAP450‐CTD displayed the highest frequency of processive transport events (Figure 4V), indicating that full‐length CDK5RAP2, together with additional binding partners, enhances the efficiency of HSET‐dependent transport. Notably, our kymographs revealed that, in addition to fusion at microtubule ends, the GFP–CDK5RAP2‐FL•AKAP450‐CTD condensates also underwent fusion and splitting events along the microtubule lattice, as indicated by the signal intensities of the diagonal lines (Figure S6N,Q). These findings support that these condensates possess dynamic properties resembling centrosomes, which undergo clustering and declustering.

GFP‐CDK5RAP2 (1‐530) presented a relatively low binding affinity for HSET, with a binding coefficient (Kd) value of ∼12 µM under Isothermal Titration Calorimetry (ITC) experimental conditions (Figure S7I). Condensate formation amplifies the localized concentration of CDK5RAP2, and the presence of microtubules increases the probability of CDK5RAP2‐HSET interactions, as both can associate with microtubules. Consequently, the observed interactions between CDK5RAP2 and HSET as condensates along microtubules are likely to occur more readily than under ITC conditions. Moreover, similar to GFP–CDK5RAP2‐FL•AKAP450‐CTD, the velocities of both GFP‐CDK5RAP2 (248‐530) and GFP‐CDK5RAP2 (1‐530) were not positively correlated with fluorescence signal intensity (Figure S7G,H). Thus, although HSET generally displays limited processivity [29, 30, 31], its processivity is significantly enhanced when the HSET motors are clustered by CDK5RAP2 condensates. Importantly, the transport speed mediated by HSET remains unaffected by increased cargo size.

### 2.7 Higher HSET Expression Levels in Cancer Cells Promote Centrosome Clustering and are Correlated With Poor Patient Prognoses

Many human cancer types—including lung squamous cell carcinoma, lung adenocarcinoma, liver hepatocellular carcinoma, and breast invasive carcinoma—exhibit elevated levels of *HSET* mRNA transcripts (Table S1). We consulted the The Cancer Genome Atlas (TCGA) and found that *HSET* mRNA levels are significantly higher in primary tumors compared to normal tissues in all examined cancers, including those mentioned above (Figure S8A–E). Moreover, consistent with previous studies [61, 62, 63], Kaplan‐Meier curves, plotted using TCGA cohort data, indicated that patients exhibiting high HSET expression have significantly poorer overall survival rates from breast, lung, and liver cancers (p = 1e‐16 to 7.1e‐08). Cox proportional hazard analysis further demonstrated that high HSET expression is a negative prognostic factor in these cancers, with hazard ratios (HR) ranging from 1.71 to 2.53 (Figure S8F–H). These results support that elevated HSET expression is significantly associated with a poor prognosis for many cancer patients. Furthermore, many cancers (e.g., breast [64] and colon [65] cancers) exhibit higher levels of HSET expression alongside centrosome amplification, indicating that increased HSET abundance contributes significantly to the clustering of extra centrosomes.

### 2.8 Mathematical Modeling Supports That HSET Mediates Centrosome Clustering

Given that (1) HSET clusters exhibit improved microtubule processivity, enabling the transport of CDK5RAP2 condensates to microtubule minus ends, and (2) elevated HSET expression levels are observed in many human cancers, next, we performed in silico simulations using Cytosim [66]. Through these simulations, we aimed to determine if higher expression levels and better processivity of HSET can promote clustering of CDK5RAP2 condensates, thereby facilitating coalescence of centrosomes in cancer cells.

First, we modified the unbinding rate of HSET in our system to simulate its processivity (Table S2). Our simulations revealed that a lower HSET unbinding rate promotes the clustering of CDK5RAP2 condensates (Figure S8I,K, Videos S3 and S4). This outcome indicates that HSET processivity enhances centrosome clustering. Previous simulations of interphase PCM assembly have indicated that ∼300 PCM complexes are required to form dynein‐mediated microtubule asters in which the PCM is concentrated at the center [67]. However, in cancer cells, centrosomes are amplified, with most cells having four centrosomes or occasionally more [11]. Our simulations further revealed that increasing the number of processive HSET molecules in the system reduces the number of CDK5RAP2 molecules required for robust clustering (Figure S8J,L, Videos S5 and S6). In contrast, fewer processive HSET molecules resulted in the formation of extensive microtubule bundles. This outcome implies that greater processivity and higher levels of HSET in cancer cells promote centrosome clustering at spindle poles, which are enriched with microtubule minus ends.

### 2.9 CDK5RAP2 Forms Condensates When Overexpressed in Mammalian Cells

**FIGURE 5:**
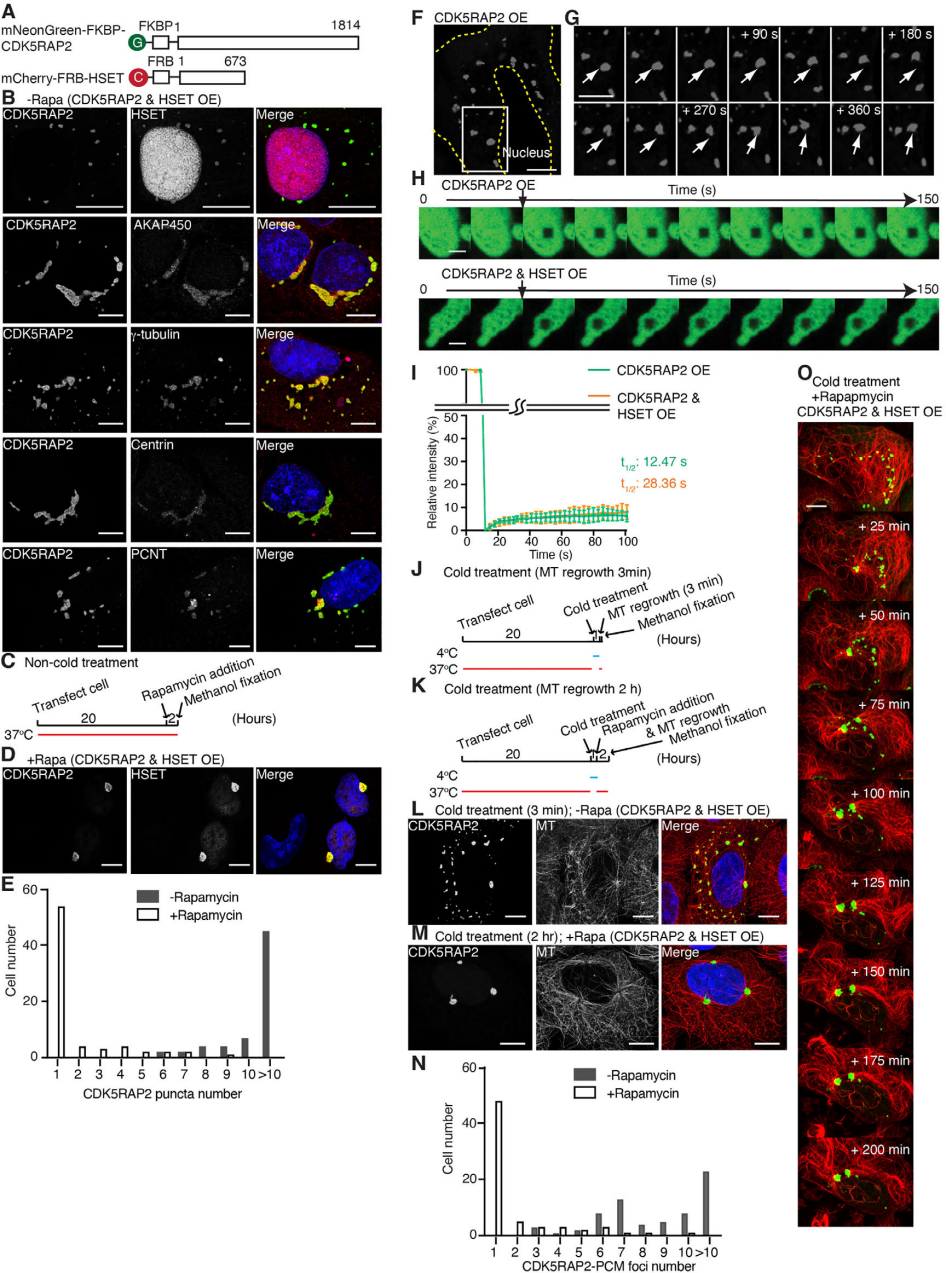
CDK5RAP2 forms PCM foci upon overexpression in mammalian cells. A) Schematic illustrations of the mNeonGreen‐FKBP plus CDK5RAP2 and mCherry‐FRB plus HSET fusion constructs used in mammalian cell lines. B) Representative immunofluorescence staining confocal images of interphase U2OS cells co‐expressing mNeonGreen‐FKBP‐CDK5RAP2 and mCherry‐FRB‐HSET in the absence of rapamycin. The panels at left show CDK5RAP2 staining, and the middle panels depict staining for HSET, AKAP450, γ‐tubulin, centrin, and PCNT. The merged fluorescence images with DAPI staining are presented in the panel at right. Scale bar: 10 µm. C) Experimental timeline of rapamycin addition without cold treatment. Each experimental step is indicated. D) Representative immunofluorescence staining confocal images of interphase U2OS cells co‐expressing mNeonGreen‐FKBP‐CDK5RAP2 and mCherry‐FRB‐HSET in the presence of rapamycin. The panels at left show CDK5RAP2 staining, and the middle panels depict staining for HSET. The merged fluorescence images with DAPI staining are presented in the panel at right. Scale bar: 10 µm. E) Histogram illustrating the distribution of numbers of CDK5RAP2 puncta during interphase in the absence or presence of rapamycin. For each condition, n > 65 interphase cells were analyzed. F) Representative confocal image of a cell expressing mNeonGreen‐FKBP‐CDK5RAP2. The yellow dashed line indicates the contours of the nucleus and cell edge. G) A montage of the boxed region in (F) is shown at a magnified scale. The numbers in the upper right corners indicate the elapsed time since initiation of time‐lapse imaging. The arrows point to one of the condensates. Scale bar: 10 µm. H) mNeonGreen‐CDK5RAP2 puncta in the cytoplasm were photo‐bleached and imaged after formation in the absence (upper row) or presence (lower row) of HSET. Scale bar: 2 µm. I) Analysis of mNeonGreen‐FKBP‐CDK5RAP2 signal in puncta after the mNeonGreen‐CDK5RAP2 puncta had been bleached in the absence (green) or presence (orange) of HSET (n  = 22). Half‐times of fluorescence recovery (*t*1/2) are indicated. J,K) Experimental timeline of cold treatment in the absence (J) or presence (K) of rapamycin. Each experimental step is indicated. L,M) Representative immunofluorescence staining confocal images of interphase U2OS cells co‐expressing mNeonGreen‐FKBP‐CDK5RAP2 and mCherry‐FRB‐HSET in the absence (L) or presence (M) of rapamycin after cold treatment. The panels at left show CDK5RAP2 staining, and the middle panels depict staining for tubulin. The merged fluorescence images with DAPI staining are presented in the panel at right. Scale bar: 10 µm. N) Histogram illustrating the distribution of numbers of CDK5RAP2‐PCM foci during interphase after cold treatment (microtubule regrowth for 2 hours) in the absence or presence of rapamycin. For each condition, n > 67 interphase cells were analyzed. O) Representative montage of confocal live‐imaging of U2OS cells co‐expressing mNeonGreen‐FKBP‐CDK5RAP2 and mCherry‐FRB‐HSET, showing GFP‐CDK5RAP2‐PCM foci (green) and SiR‐tubulin (red) dynamics following rapamycin and cold treatments. The numbers in the top right corners indicate the elapsed time since initiation of time‐lapse imaging. Scale bar: 10 µm.

Homologs of CDK5RAP2, such as *C. elegans* SPD‐5 and *Drosophila* centrosonin (Cnn), have demonstrated phase transition, thus displaying condensate‐like properties in vivo [22, 23]. In mammalian cell lines, PCM foci mediated by CDK5RAP2 during interphase and mitosis function similarly to the PCM matrix around centrioles [35, 41]. Consistently, our biochemical studies revealed the concentration‐dependent nature of GFP‐CDK5RAP2 condensate formation (Figure 3J). Therefore, we overexpressed CDK5RAP2 in U2OS cells, achieving a more than 100‐fold increase compared to endogenous levels (Figure S9G), in order to create PCM foci during interphase and assess the role of HSET in their regulation. For visualization, CDK5RAP2 and HSET were fused with mNeonGreen or mCherry at their N‐termini, respectively (Figure 5A). In addition, because HSET carries a nuclear localization signal (NLS) [32] and typically accumulates in the nucleus, CDK5RAP2 and HSET were also tagged with FKBP or FRB for spatial control, enabling enforced interaction during interphase (Figure 5A).

In the Hep3B and MDA‐MB‐231 cells, HSET localized predominantly to the nucleus during interphase due to its NLS, whereas CDK5RAP2 was mainly enriched at centrosomes in the cytoplasm (Figure S9A,B). During mitosis, both CDK5RAP2 and HSET re‐localized to the PCM and spindle poles, respectively, displaying partially overlapping signals (Figure S9C–F) [44]. To further examine their interaction in a heterologous system, we overexpressed CDK5RAP2 and HSET individually in U2OS cells (Figure S9G). Under these conditions, CDK5RAP2 formed cytoplasmic puncta, whereas HSET remained predominantly nuclear (Figure S9H,I; CDK5RAP2 and HSET are FKBP and FRB tag‐free). However, co‐expression of CDK5RAP2 and HSET led to HSET not only localizing within the nucleus, but also within the CDK5RAP2‐containing puncta in the cytoplasm (Figure 5B), confirming their interaction [44]. Furthermore, immunofluorescence staining using antibodies against centrosome proteins—including AKAP450, γ‐tubulin, centrin, and PCNT—revealed that, in the absence of rapamycin, the puncta contained all of the examined centrosome markers (Figure 5B). Interestingly, upon inclusion of rapamycin, the CDK5RAP2‐containing puncta clustered near the nucleus, leading to a reduction in the number of puncta (Figure 5C–E).

### 2.10 CDK5RAP2 Within the Condensates Formed in Cells Exhibits Limited Dynamics

We observed that the CDK5RAP2‐containing puncta appeared to be spatially confined and exhibited deformation rather than adopting a spherical shape (Figure 5F,G). FRAP analysis of the mNeonGreen‐CDK5RAP2‐hosting puncta revealed slow recovery, reaching only ∼7% of the original fluorescence intensity after photo‐bleaching (Figure 5H,I). The recovery half‐time was ∼12.5 seconds, with an immobile fraction of 93.8%. Moreover, the presence of HSET only minimally altered the dynamics of mNeonGreen‐CDK5RAP2 in these puncta (Figure 5H,I). These findings indicate that CDK5RAP2 puncta display low dynamic exchange, closely resembling the behavior of CDK5RAP2 observed in condensates in vitro and in the centrosomes of cancer cells.

### 2.11 PCM Foci Induced by CDK5RAP2 Overexpression Undergo Clustering

Given that the CDK5RAP2‐containing puncta contain γ‐tubulin, we conducted a microtubule regrowth assay after cold treatment (Figure 5J,K). The results revealed formation of microtubule asters centered on the puncta (hereafter termed CDK5RAP2‐PCM foci; Figure 5L), indicating that these puncta serve as MTOCs that nucleate the radial arrays of microtubules. Thus, since the PCM foci induced by CDK5RAP2 overexpression exhibit properties similar to centrosomes in cancer cells, they provide a useful system for investigating how HSET acts in centrosome clustering.

Upon addition of rapamycin following cold treatment, we detected a significant decrease in the number of CDK5RAP2‐PCM foci compared to conditions without rapamycin (Figure 5M,N), implying that the CDK5RAP2‐PCM foci undergo coalescence. Notably, comparable results were reproduced in non‐cancerous RPE1 cells (Figure S9J–N), demonstrating that enforced CDK5RAP2‐HSET interaction is sufficient to drive CDK5RAP2‐PCM foci clustering even in a non‐transformed cellular context. Furthermore, live‐cell imaging of individual cells expressing mNeonGreen‐CDK5RAP2 and treated with fluorescent SiR‐tubulin after cold treatment revealed a rapid transition of CDK5RAP2‐PCM foci from a dispersed to a clustered pattern (Figure 5O; Video S7). This observation indicates that centrosomes that are tethered by microtubules can move within cells, leading to their coalescence.

### 2.12 HSET Promotes Clustering of PCM Foci at the G2/M Cell Cycle Checkpoint

**FIGURE 6:**
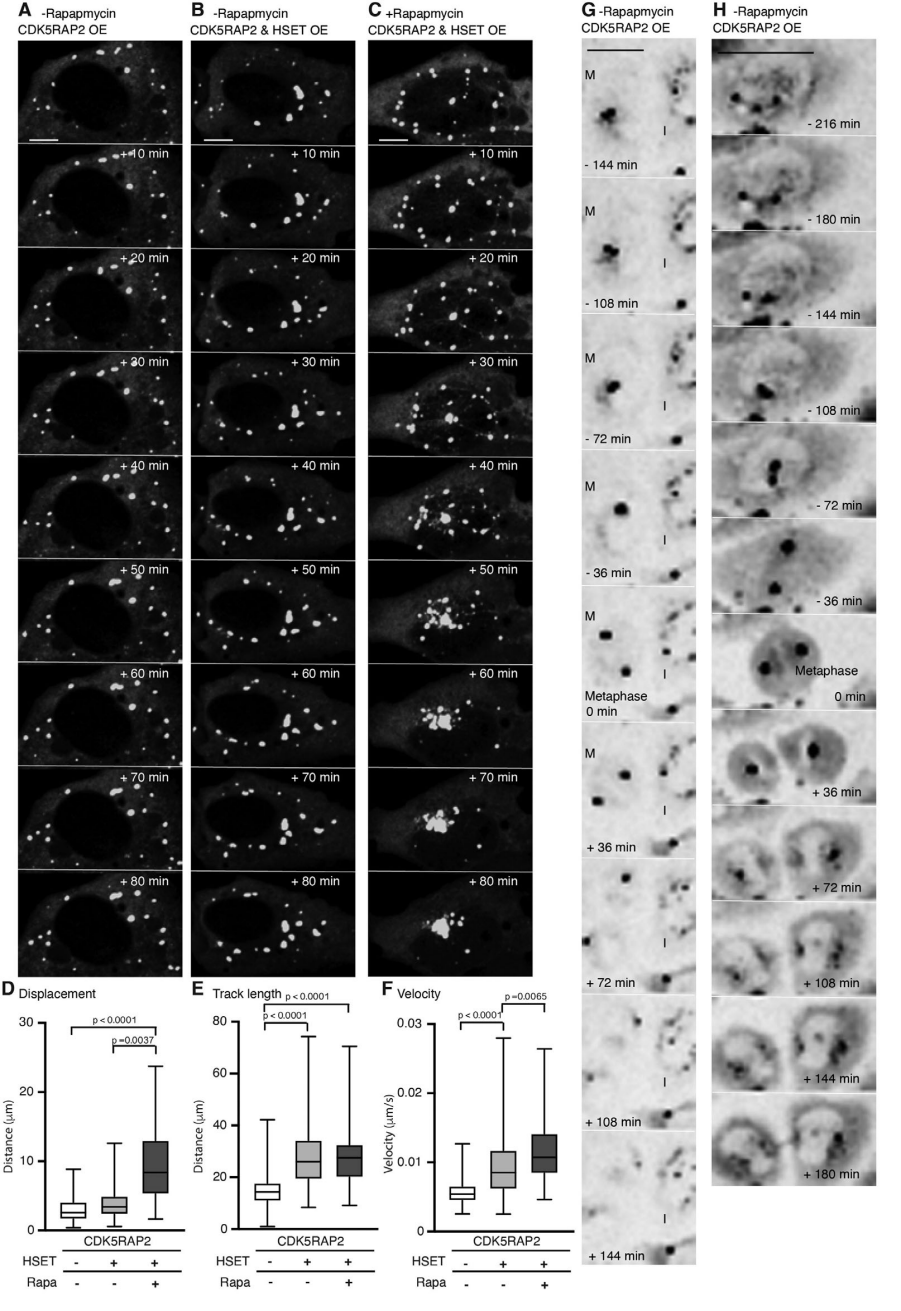
Dynamic properties of CDK5RAP2‐PCM foci throughout the cell cycle. A,B) Representative montage of confocal live‐imaging of U2OS cells expressing mNeonGreen‐FKBP‐CDK5RAP2 (A) alone or (B) together with mCherry‐FRB‐HSET, showing mNeonGreen‐CDK5RAP2‐PCM foci dynamics without rapamycin or cold treatments. The numbers in the top right corners indicate the elapsed time since initiation of time‐lapse imaging. Scale bar: 10 µm. C) Representative montage of confocal live‐imaging of U2OS cells co‐expressing mNeonGreen‐FKBP‐CDK5RAP2 and mCherry‐FRB‐HSET, showing mNeonGreen‐CDK5RAP2‐PCM foci dynamics in the presence of rapamycin without cold treatment. The numbers in the top right corners indicate the elapsed time since initiation of time‐lapse imaging. Scale bar: 10 µm. D‐F) The dynamics of CDK5RAP2‐PCM foci in live‐cell imaging under different conditions (A–C) were analyzed and plotted. Mean and standard deviation were determined from data pooled from three independent experiments (>100 condensates in total for each). Differences were assessed statistically by two‐tailed Student's *t*‐test, with *p*‐values indicated. G,H) Two representative montages of images taken from live‐imaging of HeLa cells expressing mNeonGreen‐FKBP‐CDK5RAP2 alone, showing mNeonGreen‐CDK5RAP2‐PCM foci dynamics without rapamycin and cold treatments. Mitotic and interphase cells are indicated by M and I, respectively. The cell at initiation of metaphase is indicated as the 0 min time‐point, and all other times are shown relative to it. Scale bar: 10 µm.

We further applied this experimental system to examine quantitatively the effect of HSET on the motion of the PCM foci induced by CDK5RAP2. Under the condition where no rapamycin was added and only CDK5RAP2 was expressed, PCM foci exhibited smaller displacements, shorter track lengths, and slower velocities compared to the condition where CDK5RAP2 and HSET were co‐expressed, regardless of rapamycin presence. This outcome supports that the PCM foci were significantly more confined in the absence of HSET (Figure 6A,B,D–F; Figure S10A,B, Videos S8 and S9).

HSET contains an NLS and shuttles between the nucleus and cytoplasm [32]. Rapamycin treatment enforced the interaction between CDK5RAP2 and HSET, thereby increasing the cytoplasmic pool of HSET and reducing its nuclear localization, as reflected by a ∼3.3‐fold increase in the cytoplasmic‐to‐nuclear ratio of mCherry‐HSET signals (Figure S10D–H). Consistent with this redistribution, all motion parameters of PCM foci were substantially increased in the presence of HSET and rapamycin, including larger displacements, longer track lengths, and faster velocities (Figure 6C–F; Figure S10C and Video S10). Moreover, treating cells with 5 µM of the dynein inhibitor Dynapyrazole A did not alter the dynamics of PCM foci (Figure S10I,J). Together, this rapamycin‐dependent system enforces interaction between CDK5RAP2 and HSET and thereby modulates cytoplasmic HSET concentrations, ultimately influencing the clustering behavior and motility of PCM foci.

Next, we performed live‐cell imaging on cells overexpressing CDK5RAP2 alone, without cold treatment or rapamycin, to assess if endogenous HSET facilitates clustering of CDK5RAP2‐containing PCM foci during cell division. We tracked the trajectories of PCM foci from late G2 phase (∼2 hours before metaphase) until cell division. The PCM foci barely moved in the cytoplasm during interphase (Figure 6G, interphase cell (I); Video S11). However, as cells progressed into metaphase, the PCM foci typically clustered into one or two large foci that served as spindle poles (Figure 6G; Figure S10K and Video S13). Upon mitotic exit, these large foci dispersed into small ones (Figure 6H (+36 to +180 minutes); Video S12), resembling the disassembly of clustered centrosomes observed in Hep3B cells (Figure 1I; Figure S2D). HSET expression is tightly regulated throughout the cell cycle, being upregulated as the cell enters mitosis [68] and declining upon mitotic exit in an APC/C‐dependent manner (Figure S10L) [69]. Taken together, these findings indicate that centrosome clusters undergo declustering when HSET protein levels are reduced, implicating HSET in stabilizing clustered centrosomes and maintaining centrosome integrity.

### 2.13 HSET Self‐Assembly Contributes to the Stability of Clustered Centrosomes and Prevents Declustering

**FIGURE 7:**
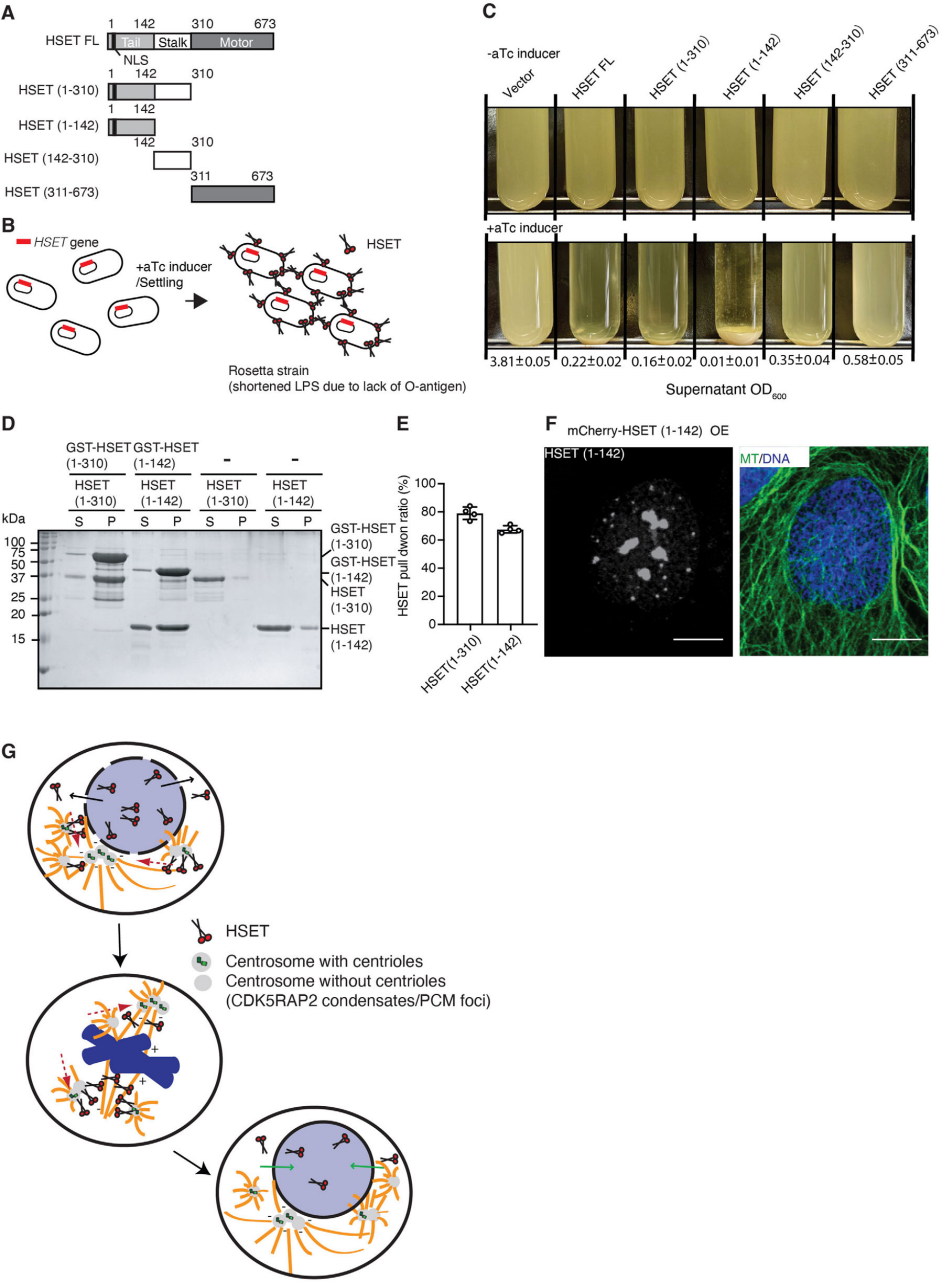
HSET stabilizes clustered centrosomes through its N‐terminal region. A) Schematic representations of the domain structure of full‐length HSET and the truncation constructs used in this study. The NLS is indicated. B) Schematic illustrating bacteria expressing either full‐length or truncated HSET (red) on their cell surface, facilitating cell‐cell adhesion. Protein expression was induced by anhydrotetracycline (aTc). C) Macroscopic aggregation analysis to assess cell‐cell adhesion. Cultures of *Escherichia coli* Rosetta strain lacking the major O‐antigen, and thus carrying a shortened LPS [79], were engineered to display full‐length or truncated HSET on their surfaces. After incubation, cultures were allowed to settle, and OD_600_ values of the supernatant (shown below the image) were measured to quantify aggregation. D) Pull‐down assays of non‐tagged HSET using GST‐tagged HSET. GST‐tagged HSET (1‐142) or HSET (1‐310) was incubated with non‐tagged HSET (1‐142) or HSET (1‐310), respectively. GST‐bound and unbound fractions were analyzed by SDS‐PAGE and stained with Coomassie blue. E) Quantification of GST‐HSET pull‐down assays. Bar graph showing the percentage of HSET band intensity in pull‐down fractions relative to supernatant fractions. F) Representative immunofluorescence staining images of interphase U2OS cells expressing mCherry‐HSET (1‐142) in the absence of rapamycin. Left panels show HSET staining; right panels show merged images with microtubules and DAPI. Scale bar: 10 µm. G) In cancers displaying elevated HSET expression, HSET leaks from the nucleus and clusters centrosomes at the onset of mitosis. During mitosis, these clustered centrosomes are positioned at the spindle poles through the microtubule binding, bundling, and cargo transport activities of HSET. At the conclusion of mitosis, HSET is degraded via the APC/C‐dependent pathway. Any remaining HSET is transported back to the nucleus during the interphase of daughter cells, leading to dispersion of the centrosomes. Centrosomes with or without centrioles are depicted.

To investigate if HSET on the surfaces of centrosomes self‐assembles to stabilize clustered centrosomes, we introduced full‐length or truncated HSET constructs into *Escherichia coli* (Figure 7A). Then, we performed a macroscopic cell aggregation assay [70, 71], using optical density (OD_600_) measurements to assess the extent of aggregation induced by the HSET displayed on the bacterial surfaces (Figure 7B). If HSET indeed self‐assembles, we anticipated observing precipitation of bacterial cultures. Indeed, we observed significantly reduced OD_600_ values for the supernatants in which bacteria expressed full‐length HSET, as well as the HSET (1‐310) and HSET (1‐142) truncation variants, compared to that of the HSET (142–310) and HSET (311–673) variants (Figure 7C). Furthermore, HSET (1‐142) elicited pronounced precipitation, indicating that HSET self‐assembly, mediated by its N‐terminal residues (1‐142), can stabilize clustered centrosomes and prevent declustering.

Moreover, we performed pull‐down assays using GST‐tagged N‐terminal regions (residues 1–142 and 1–310) of HSET to pull down non‐tagged HSET (1‐142) or HSET (1‐310), respectively (Figure 7D,E). These results indicate a direct and specific intermolecular interaction, supporting specific self‐association rather than nonspecific aggregation in bacterial culture. Next, overexpression of HSET (1‐142) in mammalian cells resulted in condensate formation in the nucleus (Figure 7F; Figure S10M), demonstrating that this self‐associating behavior also occurs in a physiological cellular context and it is not restricted to bacterial conditions. In contrast, neither HSET (1–310) nor full‐length HSET formed similar nuclear puncta (Figures S9I and S10M,N). These results are consistent with HSET (1‐142) inducing stronger bacterial aggregation compared to HSET (1‐310) and HSET FL. Thus, the N‐terminus harbors an intrinsic self‐assembly module that is negatively regulated by the C‐terminal region.

Upon HSET being degraded via the APC/C‐dependent pathway at the end of mitosis, the clustered centrosomes can then undergo declustering during interphase (Figure 7G; please also see Discussion). Additionally, since the motor domain of HSET is located at the C‐terminus, its self‐assembly via the N‐terminal region (1‐142) is ATP‐independent. These findings indicate that HSET exerts an additional function in regulating centrosome integrity that is independent of its ATPase activity.

## 3 Discussion

In healthy cells, interactions between CDK5RAP2 and HEST bridge centrosomes and spindle poles, which are crucial for spindle integrity [44]. Here, we have demonstrated that, in cancer cells, mechanical forces generated by CDK5RAP2 and HEST contribute to coalescence of extra centrosomes at the onset of mitosis, ensuring the integrity of clustered centrosomes at the spindle poles throughout the cell cycle (Figure 7G). By interacting with CDK5RAP2 that localizes to the PCM, HSET exerts this mechanical force to transport centrosomes directly along microtubules (Figure 7G). Intriguingly, we observed more dispersed centrosomes during interphase after AZ82 treatment or siRNA knockdown (Figure 2L,M). As HSET is primarily localized in the nucleus during interphase, this treatment effect may reflect perturbation of HSET's function in centrosome integrity during mitosis or other indirect pathways. Alternatively, different motors may contribute to CDK5RAP2 transport at distinct cell cycle stages. For instance, CDK5RAP2 can be transported to the centrosomes by cytoplasmic dynein [72], and this pathway may support centrosome clustering during both interphase and mitosis. However, since HSET is highly expressed in many cancer cells and is correlated with poor prognoses and overall patient survival, it is likely that the HSET‐dependent pathway contributes significantly to the clustering of supernumerary centrosomes in cancer cells.

In addition to interacting with HSET, CDK5RAP2 also associates with AKAP450, an interaction that has been shown previously to enhance overall CDK5RAP2 stability in cells [55]. Although CDK5RAP2 functions broadly to recruit γ‐TuRC to multiple MTOCs [73], including the centrosome and the Golgi, AKAP450 plays a more specialized role in regulating the Golgi localization of CDK5RAP2 and Golgi‐associated microtubule nucleation [55, 57]. Therefore, although AKAP450 contributes to CDK5RAP2 stability and Golgi‐based microtubule organization, the centrosomal localization of CDK5RAP2 and centrosome‐dependent microtubule nucleation can be governed by additional regulatory factors.

Rapamycin is widely used as an mTORC1 inhibitor. However, canonical mTORC1‐dependent biochemical effects (e.g., altered phosphorylation of downstream targets) typically require prolonged treatment. In contrast, short‐term rapamycin exposure primarily functions to induce FKBP–FRB heterodimerization. In our experimental system, rapamycin was applied for 2 hours under conditions of high expression of mNeonGreen‐FKBP‐CDK5RAP2 and mCherry‐FRB‐HSET. Under these conditions, the majority of rapamycin is expected to be sequestered by FKBP–FRB binding, thereby enforcing CDK5RAP2–HSET interaction rather than robustly inhibiting endogenous mTOR signaling. Consistently, rapamycin treatment resulted in a ∼3.3‐fold increase in the cytoplasmic‐to‐nuclear ratio of mCherry‐HSET (Figure S10H), supporting the conclusion that enforced CDK5RAP2–HSET interaction increases cytoplasmic HSET availability. Importantly, our live‐cell imaging experiments further demonstrate that CDK5RAP2 puncta in stable Hep3B cells and transiently transfected HeLa cells undergo clustering and declustering dynamics even in the absence of rapamycin (Figures 1I and 6G,H). Together, these results indicate that rapamycin functions in our scenario as a chemical dimerizer to acutely promote CDK5RAP2‐HSET interaction, rather than by modulating mTOR signaling.

Notably, CDK5RAP2 protein levels differed between the stable expression and transient transfected systems. Nevertheless, CDK5RAP2 foci in both systems underwent clustering upon mitotic entry and declustering after mitotic exit. These observations indicate that centrosome clustering and integrity are achieved when HSET levels exceed a functional threshold. Notably, HSET has been reported previously to exhibit cooperative behavior in its cellular functions (e.g., cargo transport [74]), and such cooperativity may contribute to the robustness of clustering dynamics. Importantly, increased CDK5RAP2 levels do not override HSET‐dependent constraints, indicating that HSET activity remains the limiting factor governing clustering equilibrium.

The centrosome shares several characteristics with phase‐separated condensates, including that pericentriolar proteins can undergo a process known as maturation, leading to the centrosome transitioning from a liquid‐like state to a gel‐like state over time [22]. This maturation process involves hardening of the PCM proteins, providing the centrosome with the strength to resist the physical forces it encounters [19]. Interestingly, the PCM foci formed by CDK5RAP2 also appear to be confined and undergo deformation rather than adopting a spherical shape. Additionally, CDK5RAP2 in the PCM foci displays low dynamic properties, allowing HSET to be stably clustered in the PCM foci and to transport the CDK5RAP2‐hosting PCM foci. Given HSET's multifunctional mechanisms, including active transport (ATP‐dependent) and stabilization (ATP‐independent), it provides an additional layer of regulation for controlling centrosome assembly beyond the centrosome maturation pathway. This regulatory mechanism is particularly critical for cancer cell growth.

Acting as a single motor, HSET lacks processivity and does not present uni‐directionality on microtubule filaments [29, 30, 31]. Previous studies have demonstrated that HSET is involved in sliding and bundling microtubules [32]. Here, we propose a regulatory mechanism whereby HSET's processivity is enhanced by cluster formation when bound to CDK5RAP2, which is located on the outer layer of the centrosome. The oligomerization state of another motor, kinesin‐1, has also been linked to progression of amyotrophic lateral sclerosis (ALS), a motor neuron disease [75]. Kinesin‐1 is regulated by an autoinhibitory mechanism, whereby binding of cargo induces its activity [76]. Additionally, adaptor proteins that bridge intracellular cargoes (e.g., mitochondria [77, 78]) and kinesin‐1 can enhance the latter's processivity. This increased processivity of kinesin‐1 is likely due to recruitment of multiple kinesin‐1 molecules, leading to cooperative movement [75]. Additionally, spindle formation occurs within a relatively narrow time window during the cell cycle, requiring centrosomes to reach their destinations promptly. As a result, transport must be tightly regulated. Small objects typically move more easily in the cytosol due to reduced viscous friction, which increases with object size. Interestingly, the velocity of HSET‐mediated active transport does not vary with cargo sizes, making it an ideal mechanism for transporting larger cargos, such as centrosomes.

## 4 Materials and Methods

### 4.1 Cell Culture, Plasmid Construction, and Transfection

HeLa (ATCC, CCL‐2), U2OS (ATCC, HTB‐96), and 293T (ATCC, CRL‐3216) cells were grown in DMEM (Gibco, 11995040) supplemented with 10% fetal bovine serum (Merck). MDA‐MB‐231 (BCRC, 60,425) cells were cultured in RPMI (Gibco, 11875085) supplemented with 1 mM sodium pyruvate, and 10% fetal bovine serum (Merck). Hep3B (ATCC, HB‐8064) cells were grown in MEM (Gibco, 11095072) supplemented with 1 mm sodium pyruvate and 10% fetal bovine serum (Merck). hTERT‐RPE‐1 (ATCC, CRL‐4000) cells were cultured in DMEM/F12 (Gibco, 11320033) supplemented with 10% fetal bovine serum (Merck). Genes encoding the proteins of interest were amplified by PCR using Phusion polymerase (NEB) and cloned into a mammalian expression vector under the control of a CMV promoter via the In‐Fusion cloning method (Takara). For transient transfection, cells at 70% confluency were transfected with the resulting plasmid using either Lipofectamine 3000 (Thermo Fisher Scientific) or Maestrofectin (Omics Bio), following the manufacturer's suggested protocols. Transient transfection plasmids are listed in Table S3.

### 4.2 Lentivirus Production and Infection

HEK293T cells were seeded to achieve 80–90% confluency before co‐transfection with a total of 5 µg of plasmid DNA, including a pMD.G envelope plasmid (0.25 µg), a pCMV‐dR8.91 packaging plasmid (2.25 µg), and the pLAS3W‐mStayGold‐CDK5RAP2 lentivirus construct (2.5 µg), using Maesterofectin (Omics Bio). Twenty‐four hours post‐transfection, the media was replaced with fresh media containing 1% BSA to enhance the virus production, and the viral supernatant was harvested at 24 and 48 hours, then passed through a 0.45 µm filter and stored at ‐80°C. For stable cell line generation, Hep3B and RPE1 cells were plated to 50% confluency and infected with the filtered viral supernatant mixed with 8 µg/mL polybrene for 16 hours. Successfully infected cells were selected over a one‐week period in media containing 10 µg/mL blasticidin (Gibco), with expression confirmed by both Western blot and fluorescence microscopy.

### 4.3 Immunofluorescence Analysis

Cells were fixed with ice‐cold methanol before being blocked with 2% BSA in PBS for 1 hour at room temperature. A cocktail of primary antibodies was then applied overnight at 4°C in a buffer containing 2% BSA and 0.3% Triton X‐100. The specific primary antibodies used were: anti‐HSET (Abcam, ab172620), anti‐CDK5RAP2 (Atlas Antibody, AMAb91163), anti‐tubulin (GeneTex, GTX628802), anti‐Pericentrin (Sigma, HPA032101), anti‐AKAP450 (BD, 611518), anti‐gamma‐tubulin (Invitrogen, T6557), anti‐Centrin (Merck, 04–1624), anti‐CyclinD1(Abcam, ab134175), anti‐CyclinB1(Genetex, GTX100911), anti‐CyclinA2 (Genetex, GTX103042), and anti‐p21 (Cell Signaling, 2947). Following three washes with 1X PBS containing 0.1% Tween‐20 to remove unbound antibodies, the cells were incubated for 1 hour at room temperature with the following secondary antibodies, diluted in the same buffer as the primary antibodies: Alexa Fluor 647 anti‐mouse IgG (Invitrogen, A‐31571), Alexa Fluor 488 anti‐mouse IgG (Invitrogen, A‐11001), Alexa Fluor 594 anti‐rabbit IgG (Invitrogen, A‐21207), Alexa Fluor 568 anti‐mouse IgG2b (Invitrogen, A‐21144), Alexa Fluor 647 anti‐mouse IgG2a (Invitrogen, A‐21241), and Alexa Fluor 488 anti‐mouse IgG1 (Invitrogen, A‐21121). Nuclei were counterstained with DAPI. All images were acquired using a Zeiss LSM980 with Airyscan 2 confocal microscope with a 63x oil objective. Final image processing, including maximum intensity projections, was performed with ZEN imaging software.

### 4.4 Cell Rapamycin Treatment

U2OS and RPE1 cells were seeded onto cover slips and transiently co‐transfected with mNeonGreen (mNG)‐FKBP‐CDK5RAP2 and mCherry‐FRB‐HSET. To induce dimerization, 100 nm rapamycin (LC Laboratories, R‐5000) was added to the medium, and the cells were incubated for 2 hours before fixation for standard immunofluorescence, under both with and without cold‐treatment condition. To determine if condensates contained other centrosomal proteins, U2OS cells were transiently transfected with mNG‐CDK5RAP2 overnight, followed by immunofluorescence to visualize endogenous centrosomal markers. For the microtubule aster formation assay, U2OS cells were transfected with mNG‐CDK5RAP2 overnight. Microtubules were depolymerized by incubating the cells on ice for 1 hour, and subsequent regrowth was initiated by a 5‐minute incubation at 37°C before fixation and immunofluorescence. For all experiments, Z‐stack images were acquired and converted for 3D reconstruction and analysis using Imaris (Oxford Instrument).

### 4.5 Live‐Cell Imaging

U2OS cells were seeded onto a 3.5‐cm glass‐bottomed dish (Alpha Plus) and transiently co‐transfected overnight with mNeonGreen (mNG)‐FKBP‐CDK5RAP2 and mCherry‐FRB‐HSET. For live‐cell imaging of rapamycin‐induced dimerization, cells were incubated in phenol red‐free DMEM (Gibco) containing 100 nm rapamycin (LC Laboratories). Additionally, to exclude dynein‐mediated transport, cells were pre‐treated for 1 h with 5 µm of the dynein inhibitor Dynapyrazole‐A with or without rapamycin. Imaging was conducted using a Nikon Ti‐E automatic microscope equipped with a Yokogawa spinning‐disk confocal head and an Andor iXON Ultra 888 EMCCD camera. To visualize microtubule asters within condensate clustering, cells were cultured in phenol red‐free DMEM with 400 nm SiR‐tubulin (SPIROCHROME) and subjected to cold treatment to depolymerize microtubules for 1 hour. These samples were imaged with a Zeiss LSM980 inverted confocal microscope using a Plan‐Apochromat ×63/1.45 NA oil‐immersion objective in LSM Plus confocal mode. For mitotic imaging, HeLa cells were transiently transfected with mNG‐CDK5RAP2, while the mStayGold‐CDK5RAP2 stable Hep3B cell line was used to observe multipolar spindle clustering. Both cell lines were seeded on 3.5‐cm glass‐bottomed dishes (Alpha Plus) and imaged overnight in phenol red‐free media using the spinning‐disk microscope.

Z‐stack images from all experiments were acquired, reconstructed in 3D, and analyzed using Imaris (Oxford Instrument), where condensates were modeled as spheres to quantify their movement dynamics. Track length of the condensates is defined as the cumulative distance traveled by a particle over the entire duration of tracking, calculated as the sum of distances between consecutive positions along its trajectory. Displacement is defined as the straight‐line distance between the initial and final positions of a particle over the tracking period, independent of the path taken.

### 4.6 Double Thymidine Synchronization and Western Blotting

U2OS cells were synchronized at the G1/S boundary using a double thymidine block. The cells were first treated with 2 mM thymidine for 16 hours, released for 8 hours in fresh medium, and then re‐blocked with 2 mm thymidine for a further 16 hours. Following the second release, samples were collected every hour for 8 hours. For protein extraction, cells were lysed in RIPA buffer (50 mm Tris, pH 8.0, 150 mm NaCl, 1% Triton X‐100, 0.1% SDS, 0.5% sodium deoxycholate, and EDTA‐free protease inhibitors). Extracts were cleared by centrifugation at 16 000 × g for 15 minutes. Equal amounts of protein were analyzed by SDS‐PAGE and transferred to PVDF membranes. Western blotting was performed using the following primary antibodies: anti‐CDK5RAP2 (Atlas Antibodies, AMAb91163, 1:2000), anti‐HSET (Abcam, ab172620, 1:5000), anti‐Cyclin D1 (Abcam, ab134175, 1:5000), anti‐Cyclin B1 (Abcam, ab181593, 1:2000), and anti‐GAPDH (GeneTex, GTX100118, 1:5000). Protein bands were detected using HRP‐conjugated secondary antibodies (GeneTex, GTX213110, GTX213111) and a chemiluminescence kit (Genetex, GTX14698), with band intensities quantified by ImageJ software.

### 4.7 Centrosome Size and Multipolar Spindle Percentage Quantification Under siHSET Knockdown and AZ82 Treatment

Cells were cultured to 70% confluency and transfected with 50 nm siHSET using RNAiMAX (Invitrogen) for 24 hours to achieve HSET knockdown. The cells were then re‐plated for an additional 24 hours onto coverslips for immunofluorescence analysis or a 6‐cm plate for Western blot validation of knockdown efficiency and protein expression levels of CDK5RAP2 and AKAP450. For HSET inhibition, cells were seeded onto coverslips and treated with 1.5 µm (0.1% DMSO) or 0.75 µm (0.05% DMSO) of the HSET inhibitor AZ82 for 48 hours. Immunofluorescent staining was performed on the treated cells. Z‐stack images for size determination were acquired using a Zeiss LSM980 with Airyscan 2 confocal microscope, equipped with a 40x oil objective (xy resolution is 0.076 µm/pixel, and z resolution is 0.29 µm/pixel) and operated in LSM Plus mode. For quantifying the percentage of multipolar spindles, images were captured with a Zeiss Axioscan 7 slide scanner using 20x objective. All image acquisition parameters were kept consistent across samples to ensure comparability. The raw images were then converted for 3D reconstruction and volumetric analysis of the CDK5RAP2 signal using Imaris (Oxford Instruments). Western blotting was performed using the following primary antibodies: anti‐CDK5RAP2 (Atlas Antibodies, AMAb91163, 1:2000); anti‐HSET (Abcam, ab172620, 1:5000); and anti‐AKAP450 (BD, 611518, 1:1000). Protein bands were detected using HRP‐conjugated secondary antibodies (GeneTex, GTX213110, GTX213111) and a chemiluminescence kit (Thermo Scientific 34096, A38555), with band intensities quantified using ImageJ software.

### 4.8 Recombinant Protein Expression and Purification

#### 4.8.1 Cloning and Baculovirus Production

The gene for CDK5RAP2‐FL with N‐terminal His and GFP tags was cloned into a pACEBac1 vector. A second construct, CDK5RAP2‐FL with N‐terminal His and GFP tags and a C‐terminal Strep tag, was also cloned into pACEBac1. The genes for AKAP450 (a.a. 2767–3458), GFP‐HSET, and mCherry‐HSET, each containing an N‐terminal ZZ‐tag, were likewise cloned into pACEBac1. All plasmids were transformed into DH10α cells for expression of either single or multiple proteins. Clones were selected via blue‐white screening and used for baculovirus production. Sf9 and Hi5 insect cells were used to generate the baculovirus and express the proteins. The virus was amplified for three generations at a 1:30 ratio to achieve optimal titer.

#### 4.8.2 Co‐Expression and Purification of GFP‐CDK5RAP2‐FL•AKAP450‐CTD

For co‐purification, recombinant His‐GFP‐CDK5RAP2‐FL was co‐expressed with zz‐AKAP450 (a.a. 2767–3458) in Hi5 cells at a 5:1 ratio. Cells were harvested by centrifugation at 1000 x g for 5 minutes and resuspended in Buffer A (50 mm K‐phosphate, 100 mm KCl, 2 mm MgCl_2_, 1 mm EGTA, and 1 mm β‐mercaptoethanol, pH 7.4), supplemented with 0.1 mm PMSF and EDTA‐free protease inhibitors. Following douncing, the cell lysate was clarified by centrifugation at 38,000 rpm in a 45 Ti rotor for 40 minutes. The supernatant was applied to a Rabbit‐IgG coated NHS‐column 5 mL (Cytiva), which was pre‐equilibrated with Buffer A. On‐column digestion of the His‐GFP‐CDK5RAP2‐FL protein was performed overnight using TEV protease. Peak fractions were concentrated with a Centricon (Amicon) at 1700 x g, and the protein was further purified by size‐exclusion chromatography on a Hiload Superose 6 (16/600 pg) column pre‐equilibrated with Buffer A.

#### 4.8.3 Purification of HSET Proteins

Unlabeled, GFP‐tagged, and mCherry‐tagged HSET proteins were purified using Buffer B (50 mM K‐phosphate, 300 mm KCl, 2 mm MgCl_2_, 1 mm EGTA, and 1 mm β‐mercaptoethanol, pH 7.4) via the same Rabbit‐IgG column and Hiload Superose 6 (16/600 pg) column protocol. The final purified HSET proteins were stored in a buffer supplemented with 20% sucrose.

#### 4.8.4 Purification of GFP‐CDK5RAP2‐Strep

To purify GFP‐CDK5RAP2‐Strep alone, Hi5 cells were harvested in Buffer C (50 mm Tris, pH 8.0, 1 m KCl, 2 mm MgCl_2_, 1 mm EGTA, and 3 mm DTT) supplemented with 0.1 mm PMSF and EDTA‐free protease inhibitors. After douncing and centrifugation at 38 000 rpm for 40 minutes, the supernatant was incubated with Strep‐Tactin beads (QIAGEN) for 30 minutes. Unbound proteins were removed with 10 column volumes of Buffer C. The protein was eluted with Buffer D (50 mm Tris, pH 8.0, 500 mm KCl, 2 mm MgCl_2_, 1 mm EGTA, 3 mm DTT, and 10 mm Dethiobiotin).

#### 4.8.5 Protein Analysis

The quality of all purified proteins was confirmed by SDS‐PAGE followed by Coomassie blue staining. Protein concentration was determined using the Bradford assay (Bio‐Rad). All purified proteins were snap‐frozen in liquid nitrogen and stored at ‐80°C.

CDK5RAP2 and HSET truncation variants (Table S3), each with an N‐terminal GST tag followed by a PreScission protease cleavage site, were cloned into the pGEX6p‐1 expression vector. The plasmids were transformed into the Rosetta *E. coli* strain (Novagen) for protein expression. Fusion protein expression was induced by incubating the bacterial culture overnight at 18°C with 0.5 mM IPTG. The cells were harvested by centrifugation at 6000 × g for 15 minutes at 4°C and resuspended in a buffer containing 20 mm HEPES (pH 7.4), 150 mm NaCl, 3 mm DTT, and an EDTA‐free protease inhibitor cocktail. Following cell lysis by a French press, the lysate was cleared by centrifugation at 35 000 × g for 30 minutes. The clarified supernatant was incubated with GST resin (GE Healthcare) to capture the fusion proteins. The GST‐tagged protein was eluted in a buffer containing 50 mm reduced glutathione. The GST tag was subsequently removed by cleavage with PreScission protease. The cleaved protein was further purified by ion‐exchange chromatography using a HiTrapQ or HiTrapS column and then by size‐exclusion chromatography on a Superdex 200 16/600 column. Both purification steps used a buffer containing 20 mm HEPES (pH 7.4), 100 mm NaCl, and 3 mm DTT. Protein quality and purity were confirmed by SDS‐PAGE and Coomassie blue staining. The purified proteins were concentrated and stored at ‐80°C for long‐term use. Plasmids and primers used in this study are detailed in Tables S3 and S4.

### 4.9 In Vitro Condensate Assembly and Phase Diagram

GFP‐tagged CDK5RAP2 and mCherry‐HSET proteins were purified and diluted to 600 nm in HEPES buffer (20 mm HEPES pH 7.4, 100 mm NaCl, and 3 mm DTT) or 1× BRB80 (80 mm PIPES pH6.8, 1 mm MgCl_2_, 1 mm EGTA) with or without 4% PEG3350 to induce liquid–liquid phase separation. For phase diagrams, both protein and NaCl concentrations were adjusted as indicated. All protein solutions were incubated at room temperature for approximately 5 minutes before imaging. Condensates were visualized using an inverted Zeiss LSM980 confocal microscope with a Plan‐Apochromat ×63/1.45 NA oil‐immersion objective. Images were acquired with consistent parameters to ensure a direct comparison across different conditions. Quantitative analysis of the condensates was performed using ImageJ. The number and size of the condensates were quantified using the “Analyze Particles” function. The mean fluorescence intensity of both the condensates and the surrounding dilute phase was measured to calculate the partition coefficient.

### 4.10 Total Internal Reflection Fluorescence Microscopy (TIRFM) Experiments for Microtubule Binding and Motility

Taxol‐stabilized biotin‐labeled X‐rhodamine microtubules were polymerized in the presence of GMPCPP at 37°C. Polarity‐marked microtubules were prepared using X‐rhodamine and HiLyte‐647 tubulin. Flow chambers were assembled by applying double‐sided tape strips to PEGylated glass slides, sealed with biotinylated coverslips. Chambers were coated with 0.2 mg/mL neutravidin, incubated for 5 min, and then coated with 0.2 mg/mL κ‐casein to block nonspecific binding. Biotin‐labeled microtubules were immobilized on the glass surface, followed by wash step to remove the unbound microtubules. To assess microtubule binding, the final reaction mixture, consisting of 1× BRB80 buffer (80 mm PIPES, 1 mm MgCl_2_, 1 mm EGTA pH 6.8), GFP‐CDK5RAP2, HSET constructs, and 0.2 mg/mL κ‐casein, was introduced into the chamber. TIRF fluorescence images were captured using a Nikon Eclipse microscope equipped with a Ti‐E TIRF module, a 100× 1.45 NA oil immersion objective lens, and an iXON Ultra 897 EMCCD camera (Andor Technology Ltd.). GFP was excited with a 488‐nm laser (Coherent), and X‐rhodamine with a 561‐nm laser (Cobolt).

For the motility assay, 60 nm GFP‐CDK5RAP2‐FL or 60 nm GFP‐CDK5RAP2‐FL•AKAP450‐CTD was mixed with 5 nm unlabeled HSET. For experiments involving CDK5RAP2 truncation variants, 350 nm GFP‐CDK5RAP2 (a.a. 1–530) or (a.a 248–530) was mixed with 10 nm unlabeled HSET to visualize movement of the particles on the microtubules. The reaction mixture consisted of 1x BRB80 buffer supplemented with 0.2 mg/mL κ‐casein, GFP‐CDK5RAP2 FL•AKAP450‐CTD, HSET, 1 mM ATP, and an oxygen scavenger (OS) mix (35 µg/mL catalase, 4.5 µg/mL glucose, 200 µg/mL glucose oxidase, and β‐mercaptoethanol). To determine if AZ82 inhibits HSET motility, 1.5 µm AZ82 was included in the reaction.

To visualize the HSET motility in the presence of CDK5RAP2‐FL•AKAP450‐CTD, 4.5 nm unlabeled HSET spiked with 0.5 nm GFP‐HSET, along with the 60 nm CDK5RAP2‐FL•AKAP450‐CTD, was prepared in 1× BRB80 containing 0.2 mg/mL κ‐casein and 1 mm ATP, then added to the flow chamber.

For the tricolor motility assay, mCherry‐HSET and GFP‐CDK5RAP2‐FL•AKAP450‐CTD were visualized on HiLyte‐647 labeled microtubules. The reaction mixture consisted of 1XBRB80 buffer supplemented with 0.2 mg/mL κ‐casein, 60 nM GFP‐CDK5RAP2 FL•AKAP450‐CTD, 100 nm HSET (5 nm mCherry‐HSET + 95 nm unlabeled HSET), 1 mm ATP, and OS mix. Processivity events were quantified as the number of events per microtubule length (µm) multiplied by the total acquisition time (min). Images were acquired at 5s per frame for a total duration of 20 min.

To examine the processivity of HSET alone, the final reaction mixture contained 0.2 mg/mL κ‐casein and 0.5 nM mCherry‐HSET, diluted in buffer (12 mm PIPES, 1 mm EGTA, and 1 mm MgCl_2_, pH 6.8) supplemented with 1 mm ATP and OS mix. Images were acquired at 0.2s per frame for a total duration of 0.8 min.

Kymographs of GFP‐CDK5RAP2 condensates in the presence of HSET were generated using MetaMorph Online software. The software's inbuilt functions were used to calculate microtubule velocity, length, and fluorescence intensity by drawing a line along the trajectories on the kymographs. Particle movements that drifted for more than three frame (15s) were classified as diffusive event.

### 4.11 Fluorescence Recovery After Photobleaching (FRAP) Analysis

Stable Hep3B and RPE1 cell lines expressing mStaygold‐CDK5RAP2 were cultured in 35‐mm dishes with phenol red‐free medium for FRAP experiments. For in vitro assays, protein condensates were formed in buffer containing 20 mM HEPES (pH 7.4), 100 mM NaCl, and 3 mM DTT, or in 1x BRB80. The Fluorescence Recovery After Photobleaching (FRAP) experiments were conducted on an inverted Zeiss LSM 980 confocal microscope with a Plan‐Apochromat ×63/1.45 NA oil‐immersion objective. Image acquisition was performed at a single focal plane using a low‐intensity laser to minimize photobleaching. A defined region of interest was using a 488‐nm laser (10 mW) at 10% power, reducing the fluorescence intensity by more than 50%. Fluorescence recovery was subsequently monitored over time. For each time point, the mean fluorescence intensity of the bleached area was normalized against a background region (outside the condensate) and a reference region (an unbleached condensate) to account for acquisition‐related photobleaching. The normalized data were used to calculate the mobile fraction, immobile fraction, and the half‐time of recovery (t_1/2_). Data processing and fitting of the recovery curves to a single‐exponential model were performed using GraphPad Prism.

### 4.12 Self‐Interaction Pull Down Assay

Purified GST‐tagged HSET fragments, i.e., GST‐HSET (1‐310) and GST‐HSET (1‐142), were mixed with non‐tagged HSET fragments (HSET (1‐310) or HSET (1‐142)) at a 1:1 molar ratio. The mixture was added to and incubated with Glutathione Sepharose (GE Healthcare) beads in binding buffer (20 mm HEPES pH 7.4; 50 mm NaCl; 3 mm DTT) for 30 minutes on ice. The beads were then subjected to two wash rounds to remove unbound protein, and the complexes were eluted with buffer containing 50 mm reduced glutathione. The resulting fractions were resolved by SDS‐PAGE and visualized with Coomassie Blue staining. Band intensities of the input and eluate fractions were quantified using ImageJ and plotted using GraphPad Prism.

### 4.13 Isothermal Titration Calorimetry Assays

The binding affinity between CDK5RAP2 (1‐530) and HSET (1‐310) was determined using a MicroCal PEAQ‐ITC (Malvern). Both proteins were dialyzed overnight at 4°C against a buffer consisting of 20 mm HEPES (pH 7.4), 100 mm NaCl, and 1 mM β‐mercaptoethanol to ensure consistent buffer conditions between the proteins and the experimental cell. The experiment was set up with 350 µm of CDK5RAP2 (1‐530) loaded into the syringe (40 µL) and 50 µm of HSET (1‐310) in the cell (200 mL). The titration was performed with 14 injections at a stirring speed of 750 rpm, with a spacing time of 120 seconds between each injection. The cell temperature was maintained at 25°C. The raw data were analyzed by fitting the integrated heats of injection to a single‐site binding model using MicroCal PEAQ‐ITC Analysis Software to determine the binding affinity.

### 4.14 PSIPRED and AlphaFold3 Structure Prediction

The secondary structure of the CDK5RAP2 N‐terminus (a.a. 1–530) was predicted using the PSIPRED v4.0 web server (https://bioinf.cs.ucl.ac.uk/psipred) [58, 59]. To gain further insight into the 3D organization and potential folding of this region, structural modeling was performed using AlphaFold3 (https://alphafoldserver.com) [60].

### 4.15 Gene Expression Correlation Analysis of TCGA Cancers

Data from the TCGA Pan Cancer cohort were downloaded from the UCSC Xena browser and analyzed. All data were generated using the UCSC Xena Browser (http://xena.ucsc.edu/).

### 4.16 Survival Analysis

Human survival analysis was determined using the online tool KMplot (https://kmplot.com/analysis/), a web‐based, registration‐free survival analysis tool for performing univariate and multivariate survival analysis. We used our HSET gene signature as input. Statistical significance was computed using a Cox‐Mantel log rank test.

### 4.17 Bacteria Surface Display Aggregation Assay

HSET protein fragments were displayed on the surface of *E. coli* by cloning them into pDSG323 Tet‐inducible expression plasmids, which were then transformed into Rosetta2 *E. coli*. For expression, a 4 mL overnight culture in LB broth was used to inoculate a larger 50 mL culture. This culture was grown for three hours to an optical density (OD600) of approximately 0.8. The culture was then split into two 25 mL aliquots, with one being induced with 500 ng/mL anhydrotetracycline (aTc). Both cultures were incubated overnight at 25°C. After the incubation, the cultures were transferred to test tubes and allowed to settle for one hour. A sample was carefully collected from the top 25% of each culture to analyze the surface‐displayed protein. The optical density at 600 nm (OD600) of the samples was measured using an Eppendorf BioSpectrometer.

### 4.18 Quantification and Statistical Analysis

All statistical analyses were performed using Prism (GraphPad). Comparisons between two groups were made using two‐tailed Student's t tests, whereas one‐way analysis of variance (ANOVA) with Dunnett's multiple comparison test was used for comparisons among more than two groups. Data are presented as mean ± SD, with *p*‐values detailed in the figure legends.

### 4.19 Analytical Ultracentrifugation

Sedimentation velocity‐analytical ultracentrifugation (SV‐AUC) experiments were conducted using an XL‐A analytical ultracentrifuge (Beckman Coulter, USA) hosting an AnTi60 rotor (Beckman Coulter, USA). CDK5RAP2 (a.a. 1–530) was examined in buffer comprising 50 mm K‐phosphate, 100 mm NaCl, and 1 mm β‐mercaptoethanol, pH 7.4. Data collection was performed at 50 000 rpm, 280 nm, and 20°C. Partial specific volume, buffer density, and viscosity were calculated in SEDNTERP. Data were analyzed using the standard c(s) method in SEDFIT, and the continuous c(s) distribution was plotted.

### 4.20 Computer Simulations and Analysis

Simulations were performed using Cytosim (https://gitlab.com/f‐nedelec/cytosim). We recompiled a simulator adopted from Henkin et al. [80], and added the component CDK5RAP2 (parameters and their values based on Chen et al. [67]) to the simulation configuration files. Simulations were run with a time step of 0.005 sec for a duration of 500 sec. To initiate a microtubule network, we simulated HSET‐mediated formation of microtubule bundles for the first 100 sec. CDK5RAP2 complexes were then added to observe condensate kinetics and clustering under various conditions of HSET processivity and different amounts of HSET and CDK5RAP2. Positions of CDK5RAP2 complexes were recorded at the end of simulations to quantify the clustering phenomenon.

All the parameter values are listed in Table S2. Different from the setting in Henkin et al. [80], we simulated various unbinding rates of HSET to examine the effect of different HSET processivity. Each CDK5RAP2 complex comprised one microtubule nucleation site, one HSET motor end, two molecules capable of binding to each other (mimicking nonspecific adhesive interactions between CDK5RAP2 complexes), and a weak association with microtubules (same parameter values as the wanderer in Table S2).

Here, we define clustering as the formation of CDK5RAP2 complexes with short distances (<3 µm). Accordingly, we measured the pair‐wise distances between CDK5RAP2 complexes. To quantify the clustering phenomenon, we calculated the fold change in the percentage of short‐distance CDK5RAP2 complexes between 100 and 500 sec. The relationship between clustering and features of interest (HSET processivity, amounts of HSET, and CDK5RAP2 complex) was visualized using a heatmap.

## Author Contributions

P.‐P.C., T.‐H.W., and S.‐Y.T. performed cell‐based analysis. P.‐P.C., A.S., and T.‐L.H. carried out biochemical experiments. P.‐P.C. and A.S. conducted TIRF‐based assays. C.‐C.W. and S.‐H.C. performed computer simulations. P‐P.C., J.‐Y.S., and C.‐L.L. carried out bioinformatics analysis. P.‐P.C. conducted the bacteria aggregation assay. All authors analyzed the data, discussed the results, and helped write the manuscript. K.‐C.H. directed the project and prepared the manuscript.

## Conflicts of Interest

The authors declare no conflicts of interest.

## Supporting information




**Supporting File 1**: advs74651‐sup‐0001‐SuppMat.docx.

**Supporting File 2**: advs74651‐sup‐0002‐VideoS1.avi.

**Supporting File 3**: advs74651‐sup‐0003‐VideoS2.mp4.

**Supporting File 4**: advs74651‐sup‐0004‐VideoS3.mp4.

**Supporting File 5**: advs74651‐sup‐0005‐VideoS4.mp4.

**Supporting File 6**: advs74651‐sup‐0006‐VideoS5.mp4.

**Supporting File 7**: advs74651‐sup‐0007‐VideoS6.mp4.

**Supporting File 8**: advs74651‐sup‐0008‐VideoS7.mp4.

**Supporting File 9**: advs74651‐sup‐0009‐VideoS8.mp4.

**Supporting File 10**: advs74651‐sup‐0010‐VideoS9.mp4.

**Supporting File 11**: advs74651‐sup‐0011‐VideoS10.mp4.

**Supporting File 12**: advs74651‐sup‐0012‐VideoS11.mp4.

**Supporting File 13**: advs74651‐sup‐0013‐VideoS12.mp4.

**Supporting File 14**: advs74651‐sup‐0014‐VideoS13.mp4.

## Data Availability

The data that support the findings of this study are available from the corresponding author upon reasonable request.
