# Supplementary material for: Cell Cycle‐Specific Regulation of Centrosome Clustering Dynamics in Cancer Cells by the Multifunctional Kinesin HSET
Source: Adv Sci (Weinh). 2026 Mar 6;13(26):e74651. doi: 10.1002/advs.74651 (PMC13159102; doi:10.1002/advs.74651)
Supplement: Supplementary file 1 — Supporting File 1: advs74651‐sup‐0001‐SuppMat.docx. [file ADVS-13-e74651-s004.docx]

**Supplemental Information**

**Cell cycle-specific regulation of centrosome clustering dynamics in cancer cells by the multifunctional kinesin HSET**

Po-Pang Chen^1,2,#^, Athira Saju^1,3,#^, Chia-Chou Wu^1^, Tzu-Han Weng^4^, Su-Yi Tsai^4^, Tzu-Lun Huang^1^, Jia-Ying Su^1^, Chien-Ling Lin^1^, Yu‐Chun Lin^5^, See-Yeun Ting^1^, Sheng-hong Chen^1^ & Kuo-Chiang Hsia^1,2,3,6^

^1^Institute of Molecular Biology, Academia Sinica, Taipei 11529, Taiwan

^2^Institute of Biochemistry and Molecular Biology, College of Life Sciences, National Yang-Ming Chiao-Tung University, Taipei 112304, Taiwan

^3^Molecular and Cell Biology, Taiwan International Graduate Program and National Defense Medical Center, Taiwan

^4^Department of Life Science, National Taiwan University, Taipei 10617, Taiwan

^5^Institute of Molecular Medicine, National Tsing Hua University, Hsinchu 300044, Taiwan

#Equal contribution

^6^Correspondence should be addressed to K.-C. H. (e-mail: [khsia@gate.sinica.edu.tw](mailto:khsia@gate.sinica.edu.tw))

**
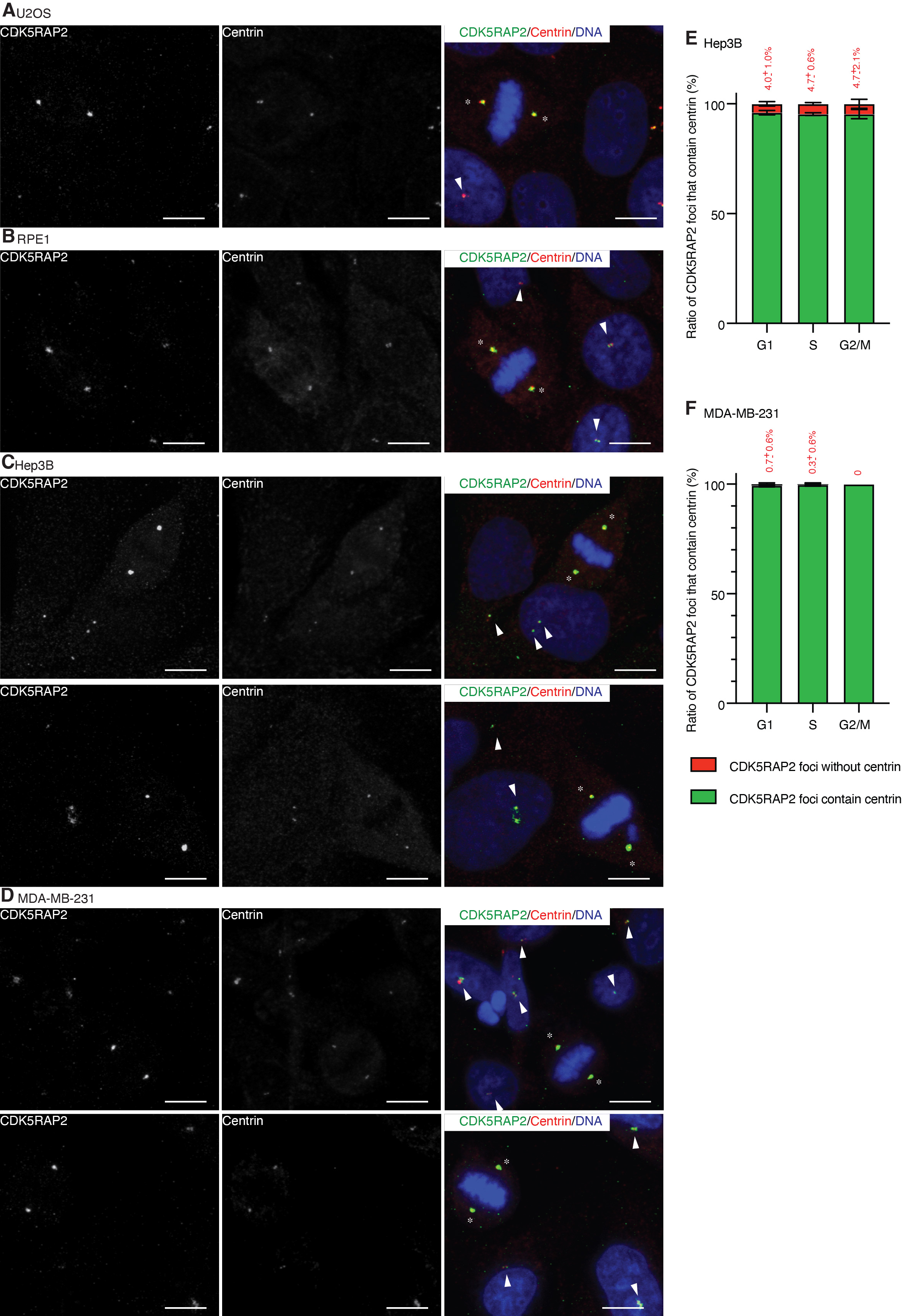
**

**Figure S1** Centrosome size varies during interphase and mitosis in liver and breast cancer cells.

A-D) Representative immunofluorescence confocal images of interphase and metaphase cells—U2OS (A), RPE1 (B), Hep3B (C) and MDA-MB-231 (D)—stained with anti-CDK5RAP2 (green) and anti-centrin (red) antibodies, and DAPI (blue). Images of the CDK5RAP2 channel (left), centrin channel (middle), and merged channels (right) are shown. Interphase and mitotic centrosomes are marked by arrows and stars, respectively. Scale bar: 10 μm. E,F) Quantification of the percentages of CDK5RAP2 foci with (red) or without (green) detectable centrin in Hep3B (E) and MDA-MB-231 (F) cells at different cell cycle stages. Mean and standard deviation were determined from data pooled from three independent experiments (*n*=300 for each cell cycle stage). Differences were assessed statistically by two-tailed Student’s t-test. The percentage of CDK5RAP2 foci without centrin is indicated in red text.

**
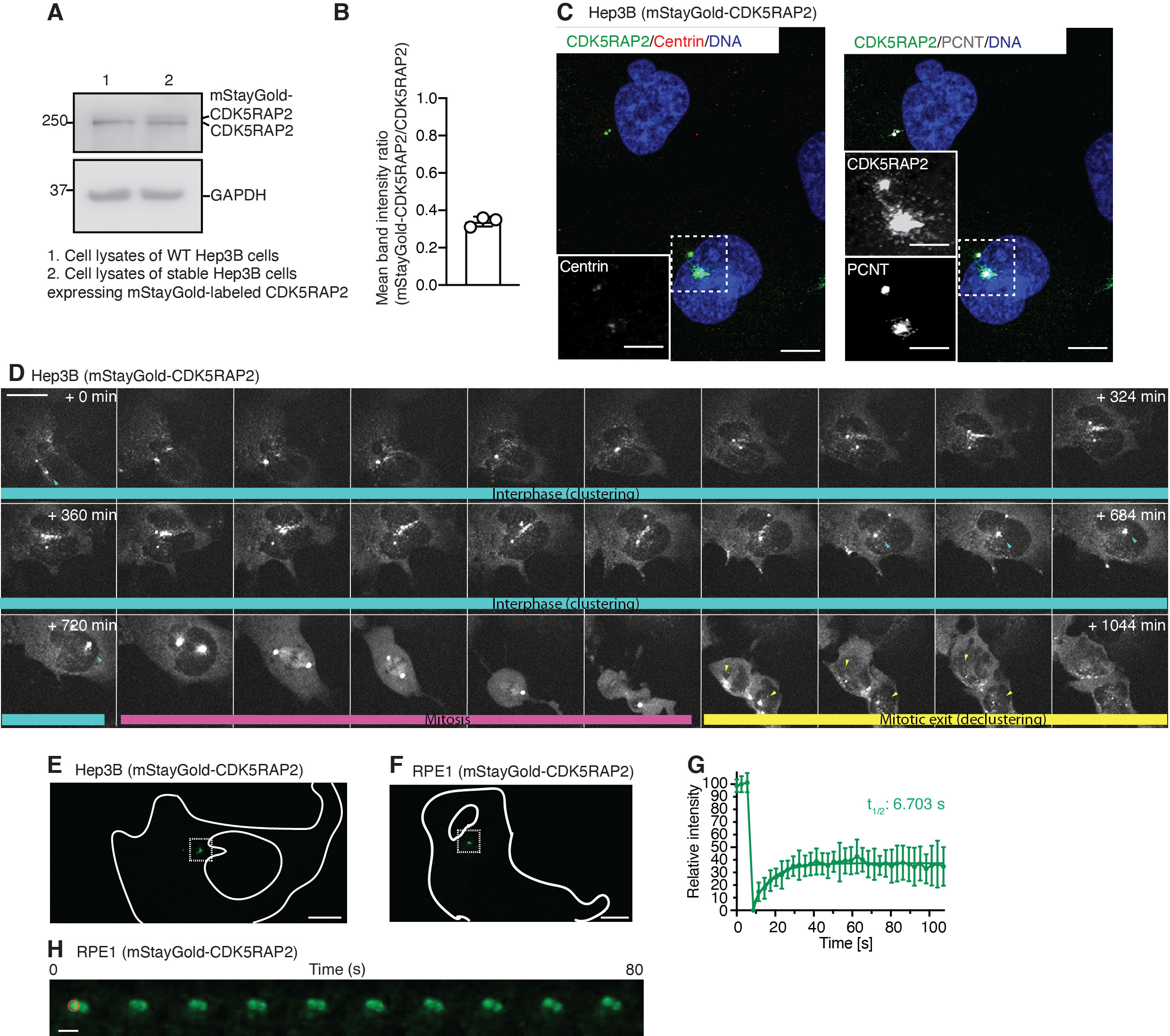
**

**Figure S2** CDK5RAP2 foci dynamics in Hep3B and RPE1 stable cell lines.

A) Western blot analysis of lysates from wild type Hep3B cells and stable Hep3B cells expressing mStayGold-CDK5RAP2, probed with anti-CDK5RAP2 antibody. B) Quantification of mStayGold-CDK5RAP2 band intensity relative to endogenous (non-tagged) CDK5RAP2 in the stable Hep3B cell line. The bar graph shows fold change. Each dot represents an individual dataset. C) Representative merged immunofluorescence confocal images of Hep3B cells expressing mStayGold-CDK5RAP2, stained with anti-CDK5RAP2, anti-centrin, and anti-PCNT antibodies, as well as DAPI. CDK5RAP2 (green), DNA (blue), and either centrin (red) or PCNT (gray) are shown in two merged images. Centrosomes within a single cell are highlighted in the boxed region and shown at higher magnification (inset). Scale bar: 10 μm; 5 μm (insets). D) Additional representative time-lapse confocal images of Hep3B cells expressing mStayGold-CDK5RAP2, recorded from late G2 through mitotic exit. Centrosomes undergoing clustering and declustering at cell cycle stages are indicated. Blue and pick arrows mark centrosomes undergoing clustering, whereas yellow arrows indicate the centrosome disassembly. The numbers in the upper right corners indicate the elapsed time since initiation of time-lapse imaging. Interphase centrosomes that underwent clustering and splitting are indicated by arrows. Scale bar: 50 μm. E,F) Representative confocal images of a Hep3B (E) or RPE1 (F) cell expressing mStayGold-CDK5RAP2. White lines delineate the nucleus and cell edge. Scale bar: 10 μm. G) Quantitative analysis of mStayGold–CDK5RAP2 fluorescence recovery after photobleaching at centrosomes (*n* = 13). The half-time of recovery (*t*1/2) is indicated. H) mStayGold-CDK5RAP2-containing centrosomes in RPE1 cells were photo-bleached and imaged. The photo-bleach site is indicated by a red circle. Scale bar: 2 μm.


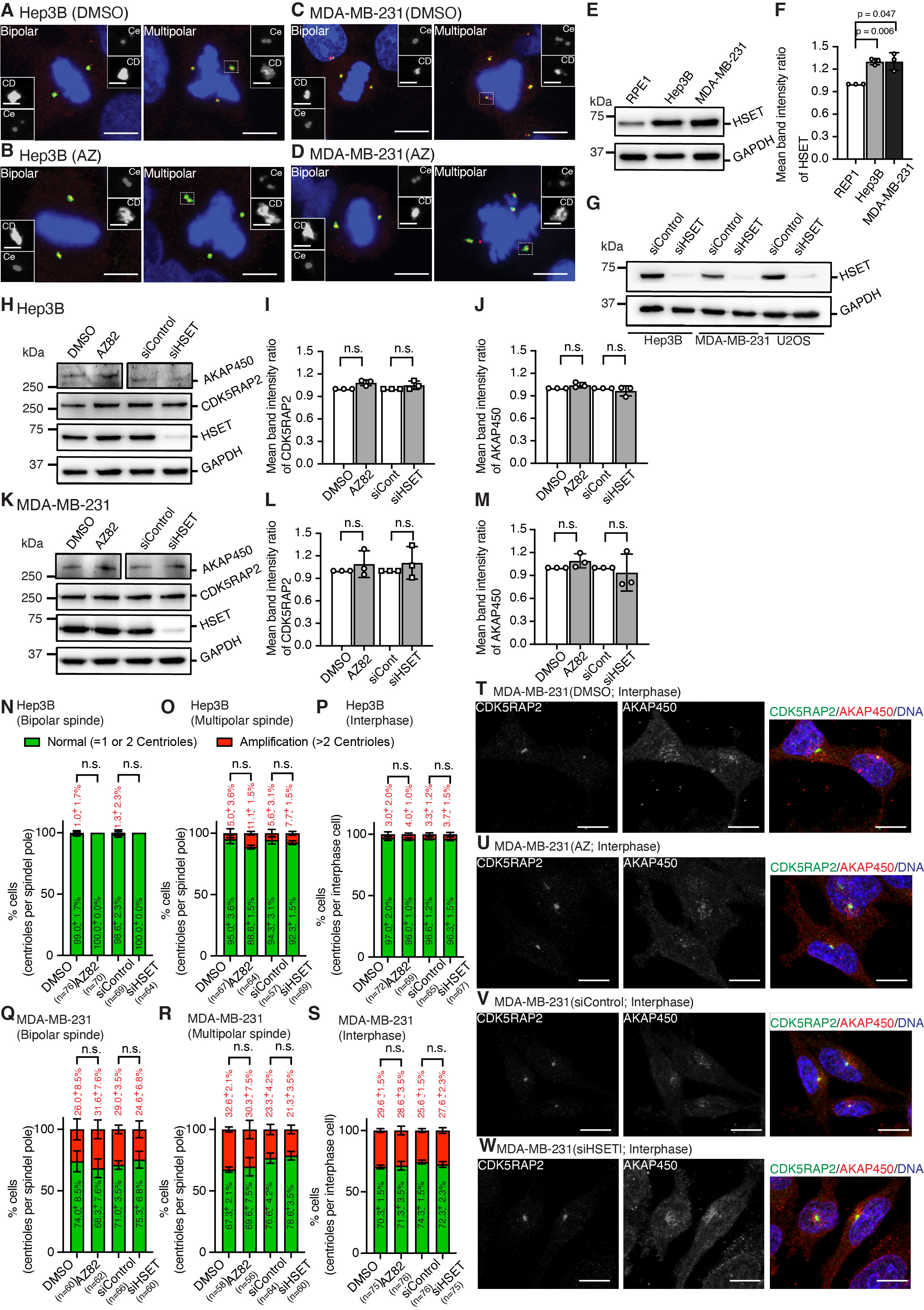


**Figure S3** HSET modulates centrosome integrity but not centriole number.

A-D) Representative immunofluorescence confocal images of bipolar and multipolar spindles in Hep3B (A,B) and MDA-MB-231 (C,D) cells treated with DMSO control or AZ82. Centrioles were stained by centrin (Ce; red), the pericentriolar material by CDK5RAP2 (CD; green), and DNA by DAPI (blue). Merged images are shown, with centrosomes at spindle poles highlighted at higher magnification (inset). Scale bar: 10 μm; 2 μm (insets). E) Endogenous HSET levels in RPE1, Hep3B and MDA-MB-231 cells were assessed by Western blotting using the indicated antibodies. GAPDH was used as a loading control. F) Quantification of endogenous HSET band intensity in Hep3B and MDA-MB-231 cells relative to RPE1 cells. The bar graph shows fold change. Statistical significance was determined by two-tailed Student’s *t*-test, with *p* values indicated. G) The Hep3B, MDA-MB-231 and U2OS cell lines were transfected with siRNA targeting HSET, and then protein levels were assessed by Western blotting using the indicated antibodies. GAPDH was used as a loading control. H) Hep3B cells were treated with AZ82 or transfected with siRNA targeting HSET, and then protein levels were assessed by Western blotting using the indicated antibodies. GAPDH was used as a loading control. I,J) Quantification of CDK5RAP2 (I) or AKAP450 (J) band intensity in AZ82-treated or siHSET-transfected Hep3B cells relative to DMSO or siControl. The bar graph shows fold change. Statistical significance was determined by two-tailed Student’s *t*-test, n.s. indicates no significant difference. K) The MDA-MB-231 cells were treated with AZ82 or transfected with siRNA targeting HSET, and then protein levels were assessed by Western blotting using the indicated antibodies. GAPDH was used as a loading control. L,M) Quantification of CDK5RAP2 (L) or AKAP450 (M) band intensity in AZ82-treated or siHSET-transfected MDA-MB-231 cells relative to DMSO or siControl. The bar graph shows fold change. Statistical significance was determined by two-tailed Student’s *t*-test, n.s. indicates no significant difference. N-S) Quantification of the percentages of Hep3B (N-P) and MDA-MB-231 (Q-S) cells with normal (1-2 centrioles) or amplified (>2 centrioles) centrosome numbers during interphase or within bipolar and multipolar spindles. Bars indicate the percentages of cells with normal (green) and amplified (red) numbers of centrosomes. *n* and percentage values are indicated. T-W) Representative immunofluorescence confocal images of interphase MDA-MB-231 cells treated with AZ82 or transfected with siRNA targeting HSET, together with corresponding controls. Cells were stained with anti-CDK5RAP2 (green), and anti-AKAP450 (red) antibodies and DAPI (blue). Images of the CDK5RAP2 channel (left), AKAP450 channel (middle), and merged channels (right) are shown. Scale bar: 10 μm.

**
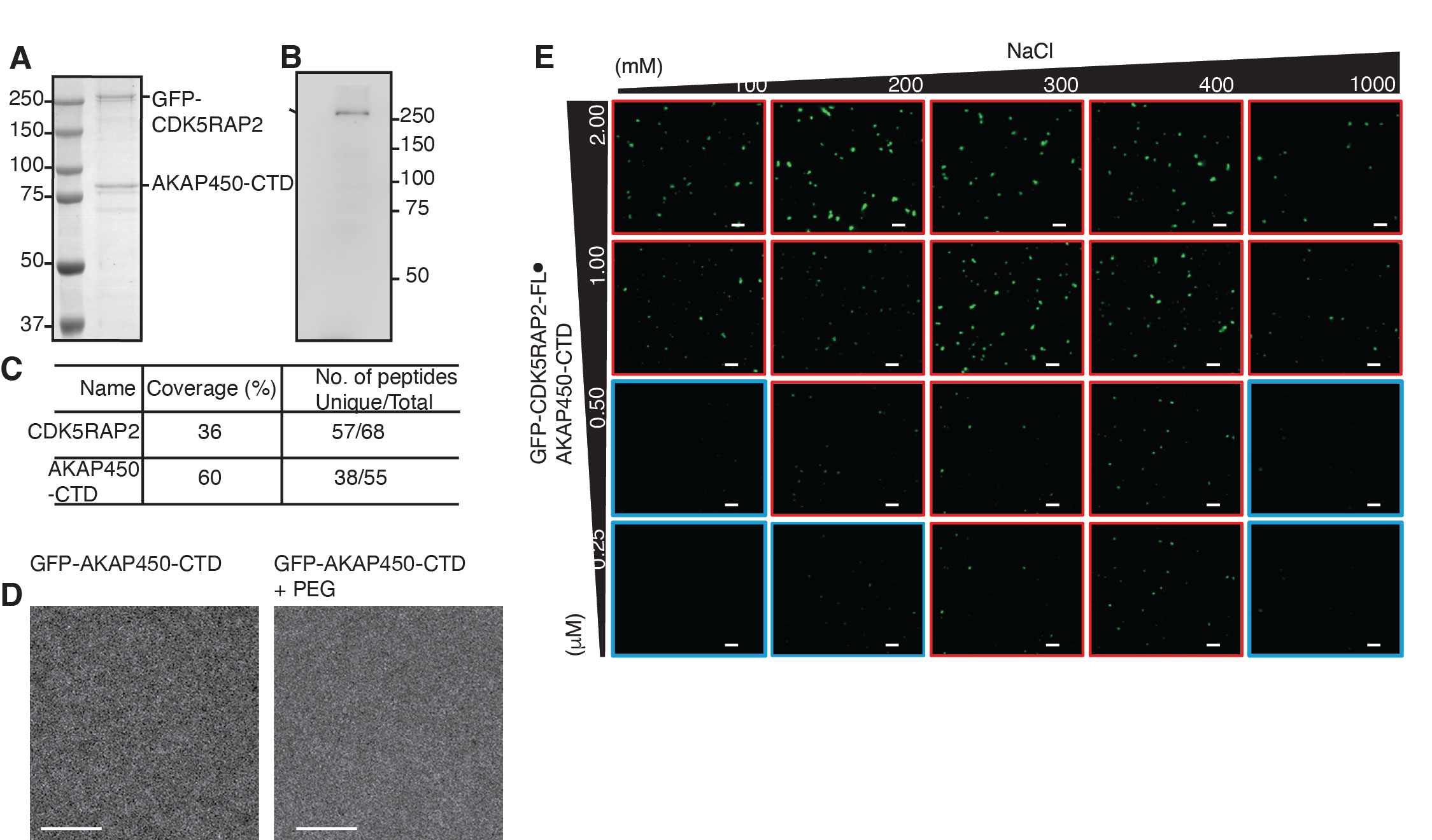
**

**Figure S4** GFP-CDK5RAP2-FL•AKAP450-CTD, but not AKAP450-CTD, forms condensates *in vitro*.

A) The purified recombinant full-length GFP-CDK5RAP2-FL•AKAP450-CTD complex was analyzed by SDS-PAGE, followed by staining with Coomassie blue. B) Western blot analysis of the complex with GFP antibody. C) Analysis of the GFP-CDK5RAP2-FL•AKAP450-CTD complex by mass spectrometry. Identities, percentage of sequence coverage, and number of peptides (unique and total) detected are shown. D) A representative TIRF image of GFP-AKAP450-CTD incubated in buffer with or without PEG and analyzed after 5 min. Scale bar: 5 μm. E) Representative TIRF images of GFP-CDK5RAP2-FL•AKAP450-CTD at the indicated protein concentrations incubated with varying salt concentrations. Scale bar: 2 μm.


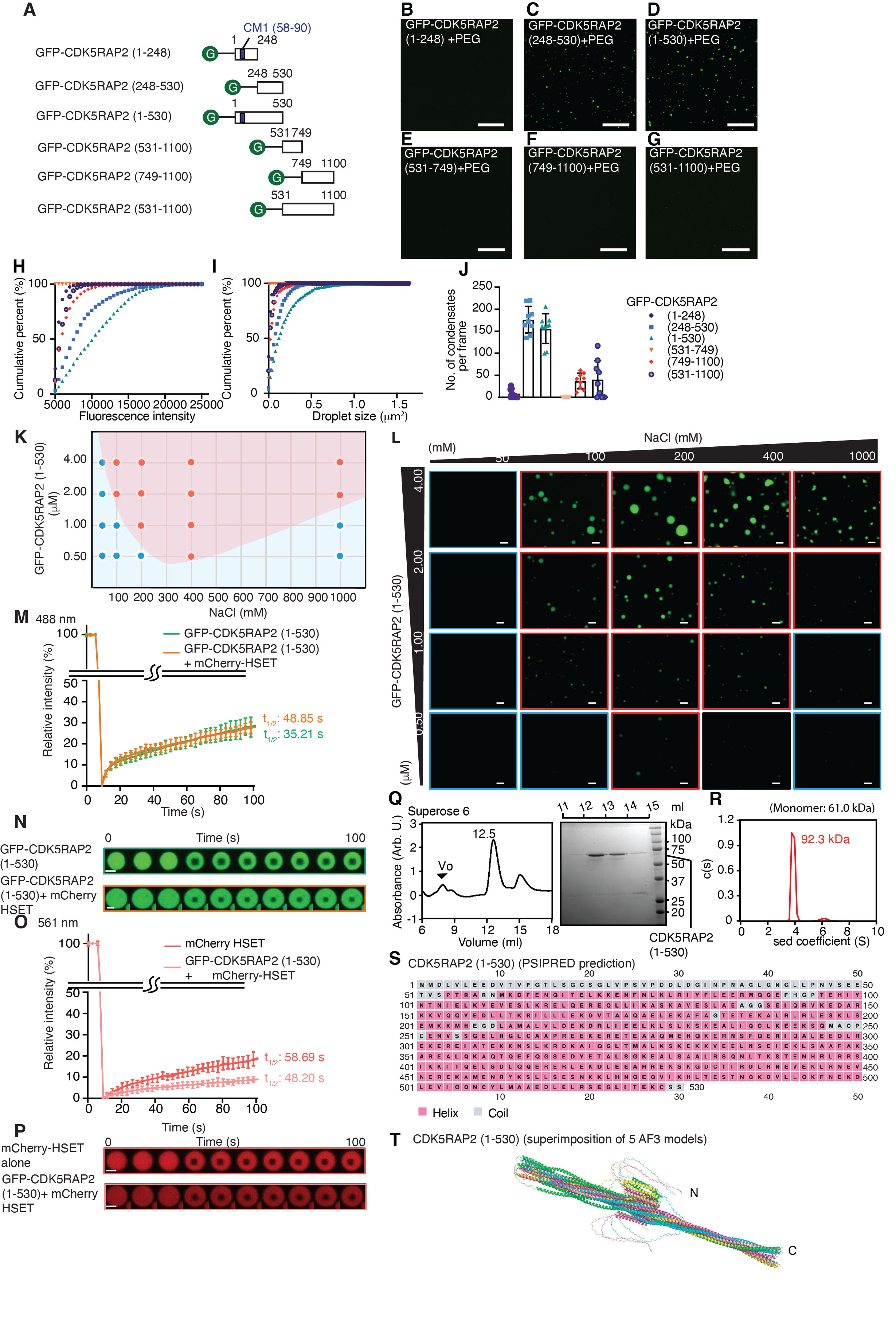


**Figure S5** The N-terminus of CDK5RAP2 facilitates condensate formation.

A) Schematic representations of the recombinant CDK5RAP2 N-terminal constructs bearing GFP at the N-terminus used in this study. The CM1 motif is indicated. B-G) Representative TIRF images of purified recombinant CDK5RAP2 N-terminal protein fragments incubated in buffer containing 4% PEG and analyzed after 5 min. Scale bar: 10 μm. H-J) The fluorescence intensity (H), droplet size (I), and droplet density (J) of condensates formed by each CDK5RAP2 N-terminal protein fragment. K) Phase diagram of the GFP-CDK5RAP2 (1-530) truncation variant under varying protein and salt concentrations. Red and blue dots indicate conditions where condensate droplets were observed or absent, respectively. The line separating the two regions indicates the approximate phase boundary. L) Representative TIRF images of GFP-CDK5RAP2 (1-530) at the indicated protein concentrations incubated with varying salt concentrations. Scale bar: 2 μm. M) Recovery kinetics of GFP-CDK5RAP2 (1-530) after bleaching of entire condensates, in the absence (green lines) or presence (orange lines) of mCherry-HSET (*n* = 18). Half-times of fluorescence recovery (*t*1/2) are indicated. N) FRAP analysis of GFP-CDK5RAP2 (1-530) condensates formed in the absence (top) or presence (bottom) of mCherry-HSET. Scale bar: 1 μm. O) Recovery kinetics of mCherry-HSET after bleaching of entire condensates, in the absence (red lines) or presence (pink) of GFP-CDK5RAP2 (1-530) (*n* = 16). Half-times of fluorescence recovery (*t*1/2) are indicated. P) FRAP analysis of mCherry-HSET condensates formed in the absence (top) or presence (bottom) of GFP-CDK5RAP2 (1-530). Scale bar: 1 μm. Q) SEC (Superose 6) elution profile of purified recombinant CDK5RAP2 (1-530) (top panel), and peak fractions analyzed by SDS–PAGE and stained with Coomassie blue (bottom panel). The void volume (Vo) of the peak fraction and absorbance (a.u.) at 280 nm are indicated. R) SV-AUC analysis of recombinant CDK5RAP2 (1-530) using the Sedfit program (red, molecular weight calculated using standards is shown in brackets). S) Protein secondary structure prediction of CDK5RAP2 (1-530) generated using PSIPRED. Residues predicted to form α-helix and coiled coil regions are shown in pink and gray, respectively. T) Superimposition of five structural models of CDK5RAP2 (1-530) predicted by AlphaFold 3.


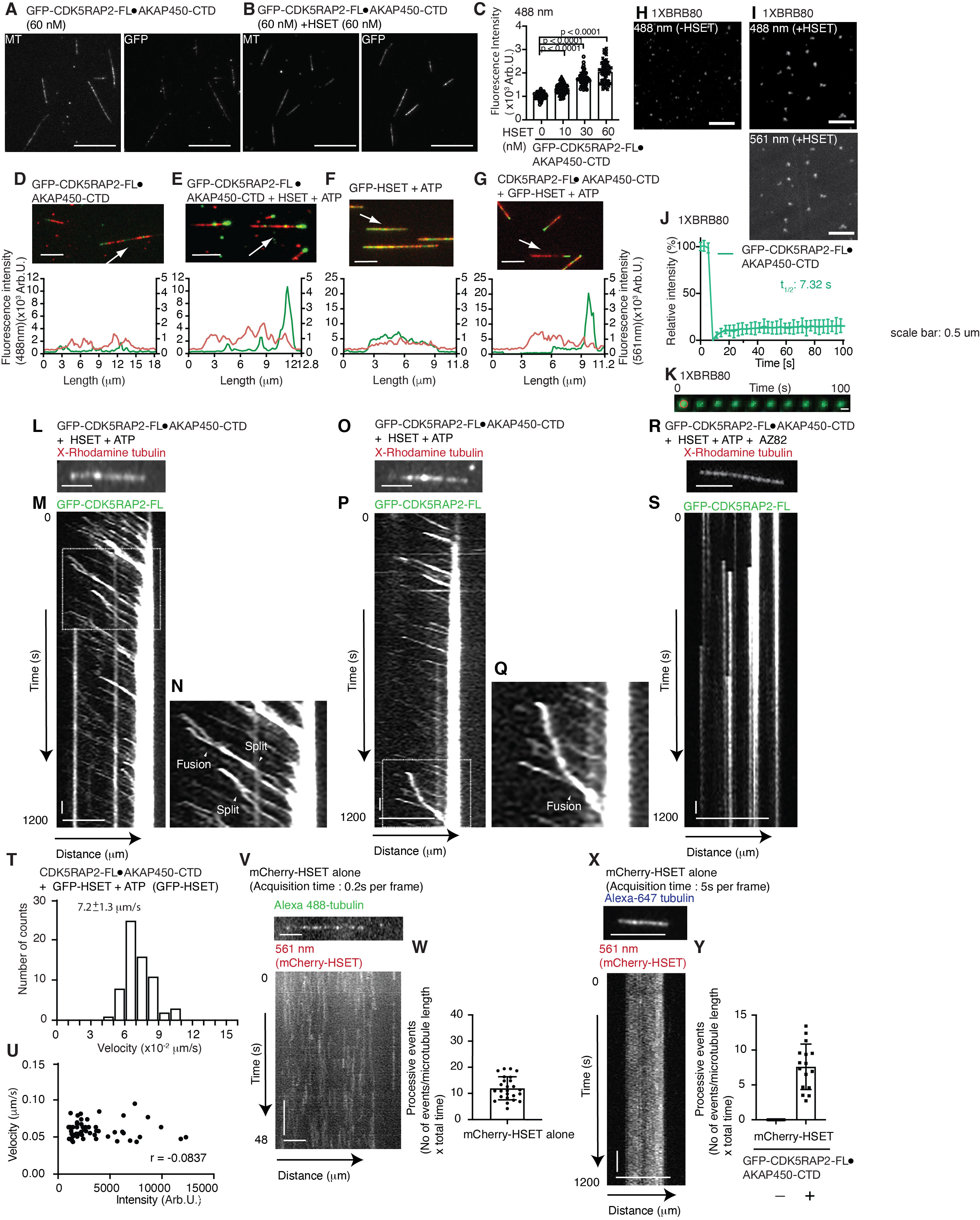


**Figure S6** HSET binds CDK5RAP2-containing condensates and transports them to microtubule ends.

A,B) Representative TIRF images of GMPCPP-stabilized microtubules (X-rhodamine- and biotin-labeled) immobilized on a glass surface incubated with GFP-CDK5RAP2-FL•AKAP450-CTD (60 nM) in the absence (A) or presence (B) of HSET (60 nM). Scale bar: 10 μm. C) Quantification of GFP-CDK5RAP2-FL•AKAP450-CTD signal intensity in the presence of the indicated HSET concentrations. Mean and standard deviation were determined from data pooled from three independent experiments (60 microtubules per measurement). Differences were assessed statistically by two-tailed Student’s t-test, with *p* values indicated. D,E) Representative merged fluorescence TIRF images of GMPCPP-stabilized microtubules (X-rhodamine- and biotin-labeled) incubated with GFP-CDK5RAP2-FL•AKAP450-CTD in the absence (D) or presence (E) of HSET and ATP (upper). Arrows indicate microtubules used for line scan analysis. Scale bar: 5 μm. Corresponding line scans along the indicated microtubules for GFP (green) and X-rhodamine (red) signals (lower). F,G) Representative merged fluorescence TIRF images of GMPCPP-stabilized microtubules (X-rhodamine- and biotin-labeled) incubated with GFP-HSET and ATP in the absence (F) or presence (G) of CDK5RAP2-FL•AKAP450-CTD (upper). Arrows indicate microtubules used for line scan analysis. Scale bar: 5 μm. Corresponding line scans along the indicated microtubules for GFP (green) and X-rhodamine (red) signals (lower). H,I) Representative TIRF images of GFP-CDK5RAP2-FL•AKAP450-CTD incubated in 1x BRB80 without PEG in the absence (H) or presence (I) of HSET. A merged fluorescence image is shown at the bottom of panel (I). Scale bar: 10 μm. J) Recovery kinetics of GFP-CDK5RAP2-FL•AKAP450-CTD in 1x BRB80 after bleaching of entire condensates (*n* = 20). Half-times of fluorescence recovery (*t*1/2) are indicated. K) FRAP analysis of GFP–CDK5RAP2-FL•AKAP450-CTD condensates formed in 1x BRB80. Scale bar: 0.5 μm. L-Q) Representative TIRF images of microtubules (L,Q) with corresponding kymographs of GFP-CDK5RAP2-FL•AKAP450-CTD (M,P) and HSET in the presence of ATP. Scale bars: 5 μm. Fusion and splitting events of GFP trajectories are boxed and shown at higher magnification (N,Q). R,S) Representative TIRF images of microtubules (R) with corresponding kymographs of GFP-CDK5RAP2-FL•AKAP450-CTD (S) and HSET in the presence of ATP and AZ82. Scale bars: 5 μm (microtubule) and 10 μm (kymograph). T) Histogram of the velocity of GFP-HSET in the presence of CDK5RAP2. Mean and standard deviation were determined from data pooled from three independent experiments (66 counts per measurement). The mean and standard deviation of velocity are shown. U**)** A scatterplot to illustrate the correlation of velocity and fluorescence signal intensity for GFP-CDK5RAP2-FL•AKAP450-CTD condensates. The Pearson correlation coefficient (r) is indicated. V-Y) Representative TIRF images of microtubules corresponding to the kymograph analysis of mCherry-HSET in the absence of GFP-CDK5RAP2-FL•AKAP450-CTD, with shorter (V) and longer (X) acquisition times. ATP is included. Scale bars: 5 μm. Quantification of events for mCherry-HSET alone with shorter (W) and longer (Y) acquisition times. The data represent a pooled analysis from three independent experiments (n = 30 and 15 for shorter and longer acquisition times, respectively). Mean values and standard deviations were analyzed statistically using a two-tailed Student’s *t*-test, with the corresponding *p*-values indicated. Processive events for mCherry-HSET in the presence of GFP-CDK5RAP2-FL•AKAP450-CTD (as shown in Fig. 4U,V) are included in (Y). Events were normalized to microtubule length and total imaging time.


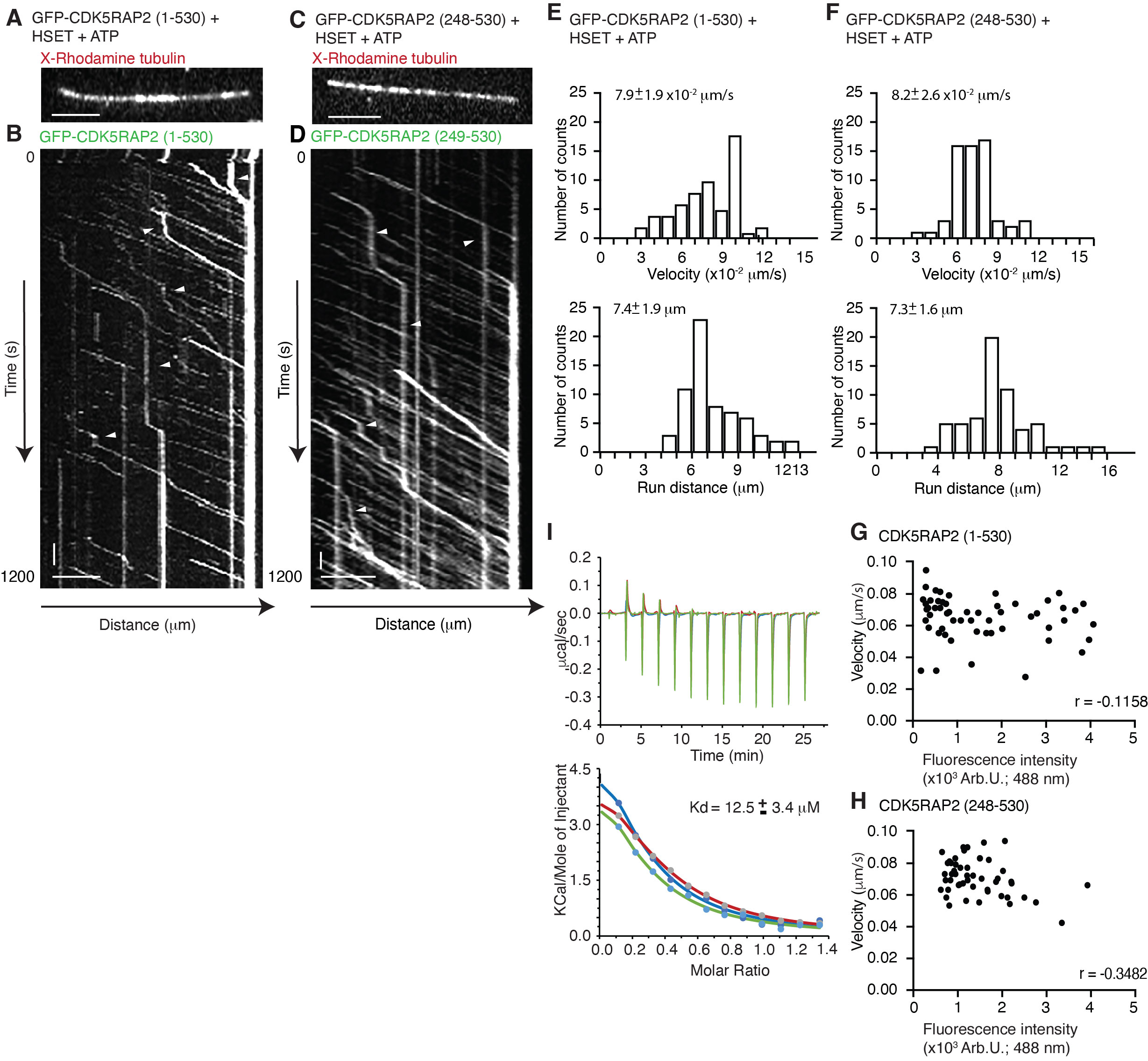


**Figure S7** HSET binds the CDK5RAP2 N-terminus and transports it to the end of microtubules.

A-D) Representative TIRF images of microtubules (A,C) with corresponding kymographs of GFP-CDK5RAP2 (1-530) (B) or GFP-CDK5RAP2 (248-530) (D) and HSET in the presence of ATP. Scale bars: 5 μm. E,F) Histograms of velocity (upper panels) and run distance (lower panels) for GFP-CDK5RAP2 (1-530) (E) and GFP-CDK5RAP2 (248-530) (F) in the presence of HSET. Mean and standard deviation were determined from data pooled from three independent experiments (59 counts in total for each measurement). Mean and standard deviation of the velocity and run distance values are shown. G,H) A scatterplot to illustrate the correlation in velocity and fluorescence signal intensity of GFP-CDK5RAP2 (1-530) (G) and GFP-CDK5RAP2 (248-530) (H) condensates. The Pearson correlation coefficients (r) are indicated. I) ITC titration curves (upper) and binding isotherms (lower) of GFP-CDK5RAP2 (1-530) with HSET (1-310). The Kd value is indicated.

**
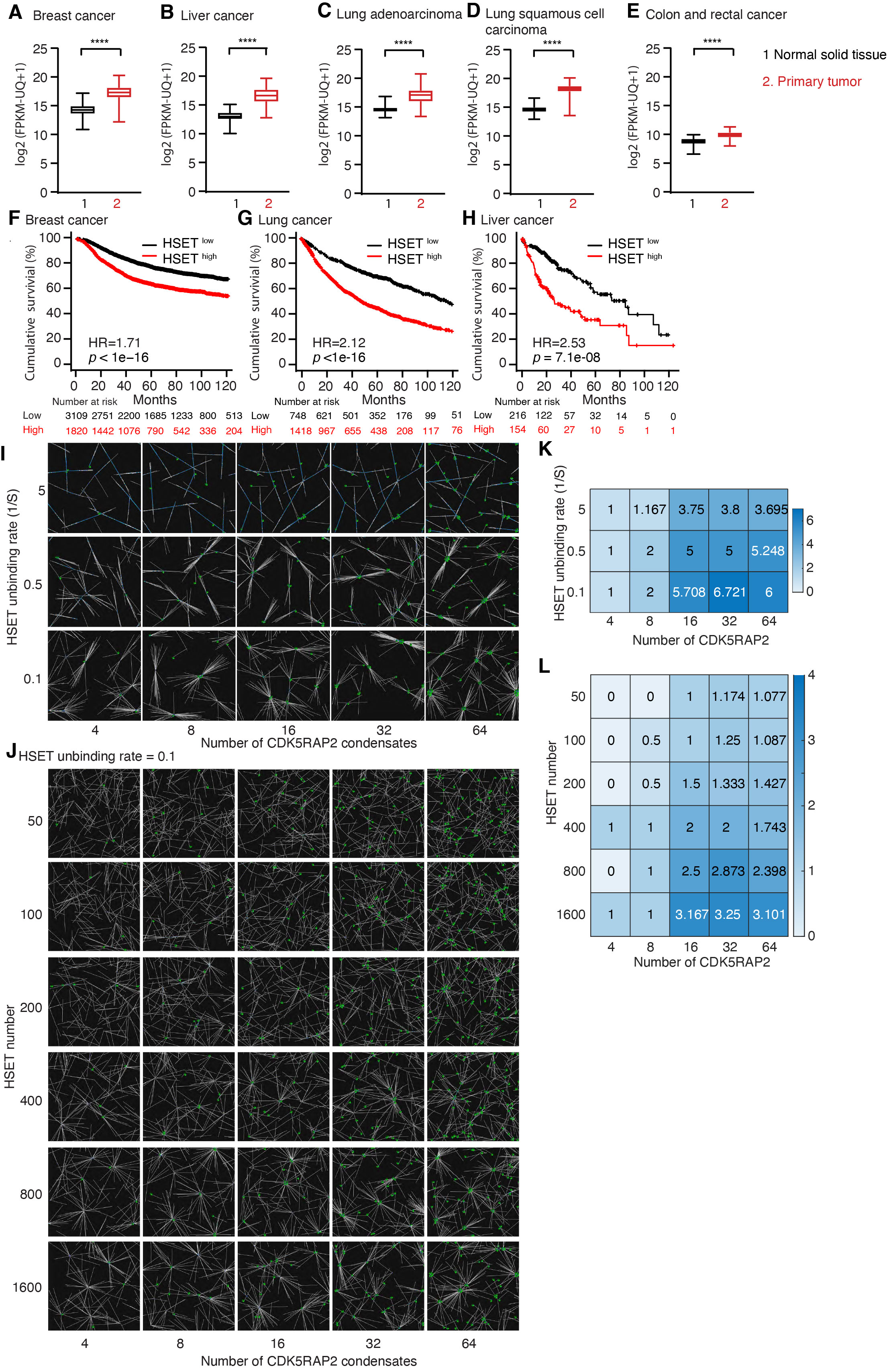
**

**Figure S8** Higher HSET expression levels and processivity promote centrosome clustering in cancer cells.

A-E) Boxplots of HSET gene expression in primary tumors of breast (A), liver (B), lung adenocarcinoma (C), lung squamous cell carcinoma (D), and colon (E) cancers relative to paired gene array data of normal solid tissue. F-H) Kaplan-Meier plots of overall survival for patients with breast (F), lung (G), and liver (H) cancers. Patients were divided into two groups (low and high) based on the median value of HSET expression. The hazard ratios (HRs) are indicated. P values with a 95% confidence indicate statistically significant variance of survival probabilities. I) Simulations of microtubules (white) with the indicated numbers of CDK5RAP2 (green) condensates and HSET unbinding rates. A consistent number of HSET molecules (4096) was used in each simulation. Green dots represent HSET.  J) Simulations of microtubules (white) with indicated numbers of CDK5RAP2 (green) condensates and numbers of HSET molecules at a fixed HSET unbinding rate of 0.1/s. Green dots represent HSET. K) A heatmap illustrating the fold change in CDK5RAP2 clustering as a function of the HSET unbinding rate (y-axis) and number of CDK5RAP2 condensates (x-axis). Darker blue indicates stronger clustering. L) A heatmap showing the fold change in CDK5RAP2 clustering as a function of numbers of HSET molecules (y-axis) and numbers of CDK5RAP2 condensates (x-axis). Darker blue indicates stronger clustering.


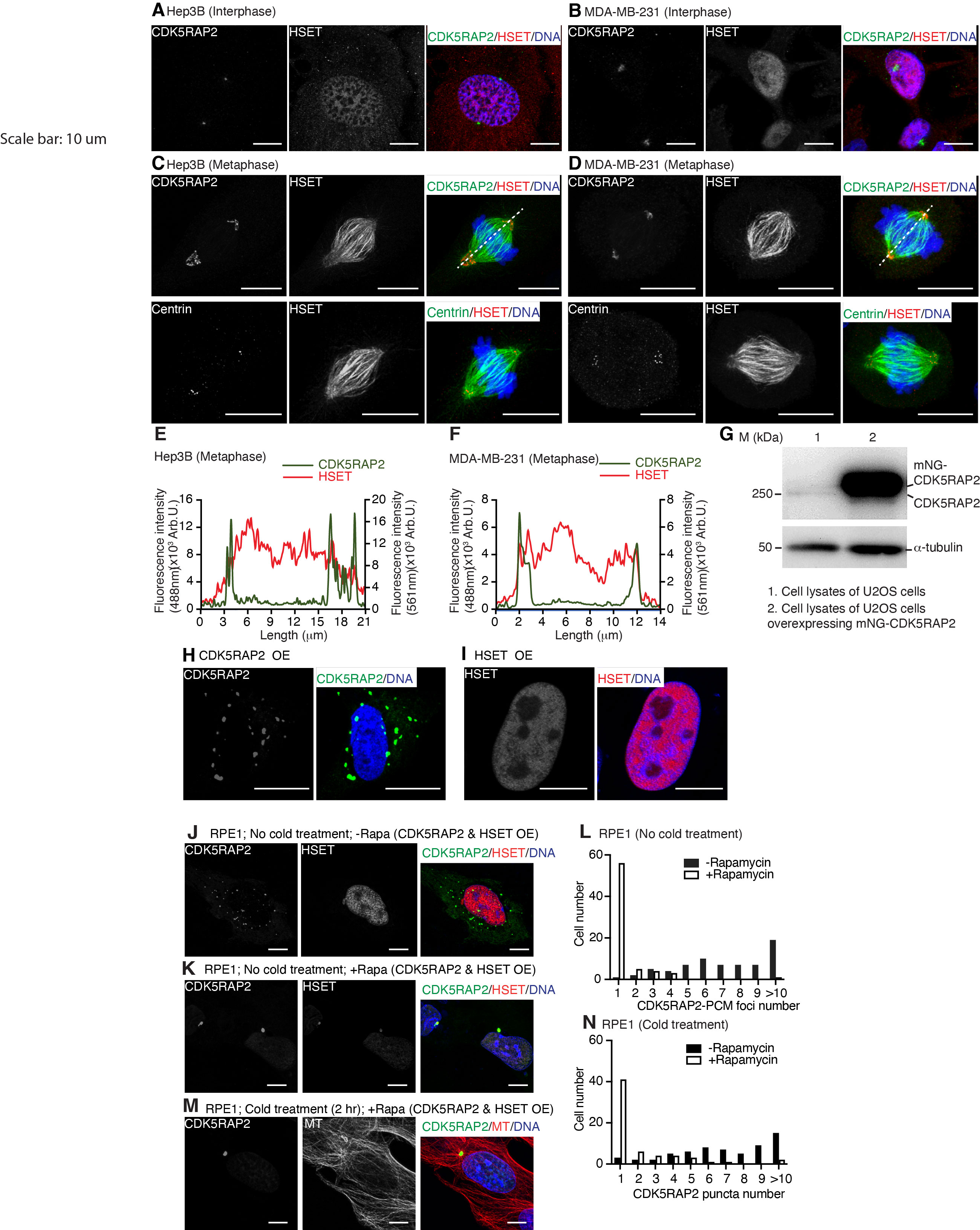


**Figure S9** Intracellular localization of CDK5RAP2 and HSET.

A,B) Representative immunofluorescence confocal images of interphase Hep3B (A) and MDA-MB-231 (B) cancer cells stained using antibodies against CDK5RAP2 (left panels) and HSET (middle panels). The merged fluorescence images with DAPI staining are shown in the panels at right. Scale bar: 10 μm. C,D) Representative immunofluorescence confocal images of mitotic spindles in Hep3B (C) and MDA-MB-231 (D) cancer cells stained using antibodies against CDK5RAP2 (upper left panels), centrin (lower left panels) and HSET (middle panels). The merged fluorescence images with DAPI staining are shown in the panels at right. Scale bar: 10 μm. E,F) Corresponding line scans along the indicated lines for CDK5RAP2 (green) and HSET (orange) in Hep3B (E) and MDA-MB-231 (F) cancer cells. G) Western blot analysis of lysates from U2OS cells and U2OS cells overexpressing mNG-CDK5RAP2, probed with anti-CDK5RAP2 antibody. α-tubulin antibody was used as a loading control. H,I) Representative immunofluorescence staining images of interphase U2OS cells individually expressing mNeonGreen-CDK5RAP2 (H) or mCherry-HSET (I) in the absence of rapamycin. The panels at left show CDK5RAP2 or HSET staining, and the merged fluorescence images with DAPI staining are presented in the panel at right. Scale bar: 10 μm. J,K) Representative immunofluorescence staining confocal images of interphase RPE1 cells co-expressing mNeonGreen-FKBP-CDK5RAP2 and mCherry-FRB-HSET in the absence (J) or presence (K) of rapamycin without cold treatment. The panels at left show CDK5RAP2 staining, and the middle panels depict staining for HSET. The merged fluorescence images with DAPI staining are presented in the panel at right. Scale bar: 10 μm. L) Histogram illustrating the distribution of numbers of CDK5RAP2 puncta during interphase in the absence or presence of rapamycin. For each condition, n > 65 interphase cells were analyzed. M) Representative immunofluorescence staining confocal images of interphase RPE1 cells co-expressing mNeonGreen-FKBP-CDK5RAP2 and mCherry-FRB-HSET in the presence of rapamycin after cold treatment. The panels at left show CDK5RAP2 staining, and the middle panels depict staining for tubulin. The merged fluorescence images with DAPI staining are presented in the panel at right. Scale bar: 10 μm. N) Histogram illustrating the distribution of numbers of CDK5RAP2-PCM foci during interphase after cold treatment (microtubule regrowth for 2 hours) in the presence of rapamycin. For each condition, n > 62 interphase cells were analyzed.


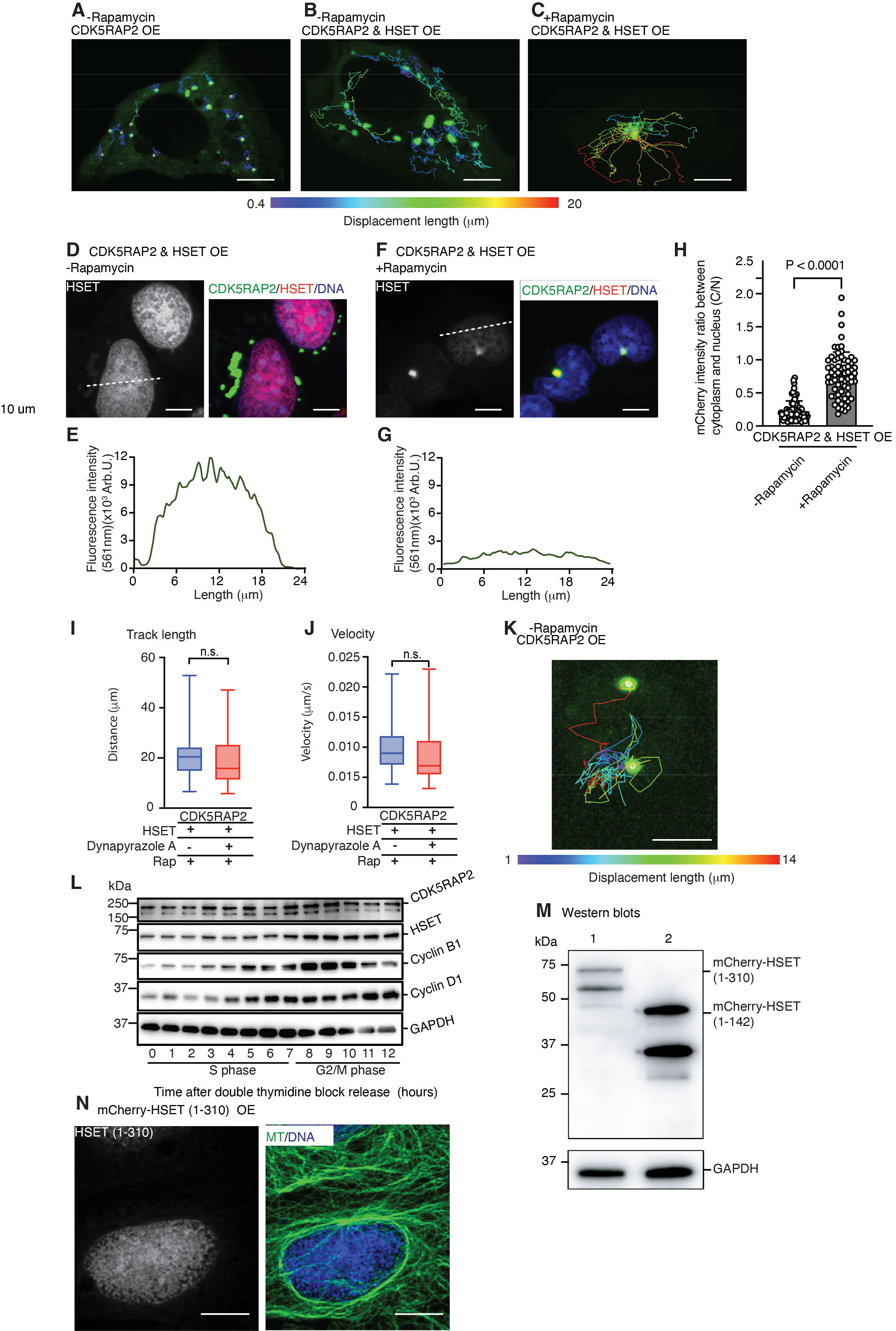


**Figure S10** Intracellular movement of CDK5RAP2-PCM foci during the cell cycle.

A,B) Representative 2D cellular migration trajectories of mNeonGreen-CDK5RAP2-PCM foci in cells expressing mNeonGreen-FKBP-CDK5RAP2 alone (A) or together with mCherry-FRB-HSET (B), showing mNeonGreen-CDK5RAP2-PCM foci dynamics without rapamycin addition. Color represents displacement length. Scale bar = 10 μm. C) Representative 2D cellular migration trajectories of mNeonGreen-CDK5RAP2-PCM foci in cells expressing mNeonGreen-FKBP-CDK5RAP2 and mCherry-FRB-HSET in the presence of rapamycin. Color represents displacement length. Scale bar: 10 μm. D-G) Representative immunofluorescence staining confocal images of cells co-expressing mNeonGreen-FKBP-CDK5RAP2 and mCherry-FRB-HSET in the absence (D) or presence (F) of rapamycin. Left panels show HSET signals; right panels show merged images including CDK5RAP2 and DAPI staining. Scale bar: 10 μm. Line-scan analyses of HSET fluorescence intensity along the indicated lines in the absence (E) or presence (G) of rapamycin are shown. H) Quantification of the cytoplasmic-to-nuclear ratio of HSET fluorescence intensity in the absence or presence of rapamycin. Statistical significance was determined by two-tailed Student’s *t*-test, *p* value is indicated. I,J) Live-cell imaging of mNeonGreen-CDK5RAP2-PCM foci in the presence of HSET and rapamycin with or without dynapyrazole-A treatment was analyzed and plotted to demonstrate their track length (I) and velocity (J). Mean and standard deviation were determined from data pooled from three independent experiments (>100 condensates in total for each). Differences were assessed statistically by two-tailed Student’s t-test; n.s.: not significant. K) Representative 2D cellular migration trajectories of mNeonGreen-CDK5RAP2-PCM foci in cells expressing mNeonGreen-FKBP-CDK5RAP2 only. Color represents displacement length. Scale bar: 10 μm. L) Cells were released from double thymidine block and harvested at the indicated times. Cell lysates were then analyzed by Western blot with the indicated antibodies. M) Western blot analysis of lysates from cells overexpressing mCherry-HSET (1-310) (lane 1) or mCherry (1-142) (lane 2), probed with anti-mCherry antibody. GAPDH antibody was used as a loading control. N) Representative immunofluorescence staining confocal images of cells overexpressing mCherry-HSET (1-310). Left panels show HSET signals; right panels show merged images with tubulin and DAPI staining. Scale bar: 10 μm.

**Supplemental Video 1**

Live-cell confocal imaging of stable Hep3B cell lines expressing mStayGold-labeled CDK5RAP2. Two mitotic cells are shown in the Figure 1I and Figure S1G.

**Supplemental Video 2**

Fluorescence time-lapse imaging of GMPCPP-stabilized microtubules (X-rhodamine- and biotin-labeled) being incubated with GFP-CDK5RAP2-FL, HSET and ATP, demonstrating that the transport process is initiated at the microtubule tip and expands toward the opposite end of the filament.

**Supplemental Video 3**

Simulation of microtubules (white) with CDK5RAP2 condensates (n=4, green) and a HSET unbinding rate of 5/s.

**Supplemental Video 4**

Simulation of microtubules (white) with CDK5RAP2 condensates (n=64, green) and a HSET unbinding rate of 0.1/s.

**Supplemental Video 5**

Simulation of microtubules (white) with CDK5RAP2 condensates (n=4, green), HSET (n=50 molecules), and a HSET unbinding rate of 0.1/s.

**Supplemental Video 6**

Simulation of microtubules (white) with CDK5RAP2 condensates (n=64, green), HSET (n=1600 molecules), and a HSET unbinding rate of 0.1/s.

**Supplemental Video 7**

Live-cell focal imaging of a single cell expressing mNeonGreen-FKBP-CDK5RAP2 and treated with fluorescent SiR-tubulin after cold treatment.

**Supplemental Video 8**

Live-cell confocal imaging of a single cell expressing mNeonGreen-FKBP-CDK5RAP2 alone (without cold treatment or rapamycin addition).

**Supplemental Video 9**

Live-cell confocal imaging of a single cell expressing mNeonGreen-FKBP-CDK5RAP2 and mCherry-FRB-HSET (without cold treatment or rapamycin addition).

**Supplemental Video 10**

Live-cell confocal imaging of a single cell expressing mNeonGreen-FKBP-CDK5RAP2 and mCherry-FRB-HSET in the presence of rapamycin (without cold treatment).

**Supplemental Video 11**

Live-cell confocal imaging of dividing cells expressing mNeonGreen-FKBP-CDK5RAP2 without HSET expression or rapamycin addition (a cell in interphase is also shown).

**Supplemental Video 12**

Live-cell confocal imaging of a single dividing cell expressing mNeonGreen-FKBP-CDK5RAP2 without HSET expression or rapamycin addition.

**Supplemental Video 13**

A zoomed-in view of the single cell shown in Supplemental Video12 expressing mNeonGreen-FKBP-CDK5RAP2 and undergoing mitosis.

**Supplemental Table 1| Fold-change in HSET expression levels between cancers and normal samples***

| **No** | **Cancer types** | **Log2 fold-change** |
| --- | --- | --- |
|  | Cervical squamous cell carcinoma | 5.77 |
|  | Uterine carcinosarcoma | 5.39 |
|  | Glioblastoma multiforme | 4.74 |
|  | Cholangiocarcinoma | 4.17 |
|  | Uterine corpus endometrial carcinoma | 3.94 |
|  | Pancreatic adenocarcinoma | 3.69 |
|  | Lung squamous cell carcinoma | 3.68 |
|  | Ovarian serous cystadenocarcinoma | 3.57 |
|  | Liver hepatocellular carcinoma | 3.34 |
|  | Bladder urothelial carcinoma BCLA | 2.97 |
|  | Brain lower-grade glioma | 2.91 |
|  | Breast invasive carcinoma | 2.83 |
|  | Lung adenocarcinoma | 2.67 |
|  | Esophageal carcinoma | 2.66 |
|  | Skin cutaneous melanoma | 2.65 |
|  | Adrenocortical carcinoma | 2.00 |
|  | Kidney renal clear cell carcinoma | 2.00 |
|  | Stomach adenocarcinoma | 1.90 |
|  | Head and neck squamous cell carcinoma | 1.46 |
|  | Colon adenocarcinoma | 1.34 |
|  | Rectum adenocarcinoma | 1.34 |
|  | Kidney renal papillary cell carcinoma | 1.19 |
|  | Testicular germ cell tumors | 1.14 |
|  | Prostate adenocarcinoma | 1.12 |
|  | Thyroid carcinoma | 0.93 |
|  | Pheochromocytoma and paraganglioma | 0.63 |

***** Calculated according to OncoDB, a database for analyzing gene expression in cancer^1^

**Supplemental Table 2| Parameters used in the Cytosim simulations.**

|  | **Value** | **Reference** |
| --- | --- | --- |
| **Cell** |  |  |
| Cell dimension | 40 μm x 40 μm x 0.2 μm | Henkin et al.^2^ |
| Viscosity | 0.02 pN⋅s/μm^2^ | Henkin et al.^2^ |
|  |  |  |
| **HSET** |  |  |
| Binding rate | 5/s | Henkin et al.^2^ |
| Binding range | 0.16 pN⋅s/μm^2^ | Henkin et al.^2^ |
| Unbinding rate | [0.1, 0.5, 5]/s | Henkin et al.^2^ |
| Unbinding force | 5 pN | Henkin et al.^2^ |
| Force-free velocity | 0.08 μm/s | Henkin et al.^2^ |
| Stall force | 5 pN | Henkin et al.^2^ |
|  |  |  |
| **Wanderer** |  |  |
| Binding rate | 0.5/s | Henkin et al.^2^ |
| Binding range | 0.16 μm | Henkin et al.^2^ |
| Unbinding rate | 0.1/s | Henkin et al.^2^ |
| Unbinding force | 5 pN | Henkin et al.^2^ |
| Diffusion | 0.1 μm^2^/s | Henkin et al.^2^ |
|  |  |  |
| **Microtubule** |  |  |
| Rigidity | 30 pN⋅μm^2^ | Henkin et al.^2^ |
| Growing force | 1.67 pN | Henkin et al.^2^ |
| Growing speed | 0.03 μm/s | Henkin et al.^2^ |
| Initial number | 128 | Henkin et al.^2^ |
|  |  |  |
| **Adhesive interaction of CDK5RAP2 complexes** |  |  |
| Binding rate | 1/s | Chen et al.^3^ |
| Binding range | 0.1 μm | Chen et al.^3^ |
| Unbinding rate | 0.5/s | Chen et al.^3^ |
| Unbinding force | 3 pN | Chen et al.^3^ |

**Supplemental Table 3| Plasmids used in this study.**

| Expression vector | Encoded protein |
| --- | --- |
| Bacterial expression | |
| pGEX-6P-1 | EGFP-CDK5RAP2 (1-248) |
|  | EGFP-CDK5RAP2 (1-530) |
|  | EGFP-CDK5RAP2 (248-530) |
|  | EGFP-CDK5RAP2 (531-749) |
|  | EGFP-CDK5RAP2 (749-1100) |
|  | EGFP-CDK5RAP2 (531-1100) |
|  | EGFP-HSET (1-310)  GST-HSET (1-310)  GST-HSET (1-142) |
| Insect cell (Hi5) expression | |
| pACEBac1 | EGFP-CDK5RAP2 FL |
|  | CDK5RAP2 FL |
|  | EGFP-HSET FL |
|  | mCherry-HSET FL |
|  | HSET FL  AKAP450 (2767–3458) |
| Mammalian cell expression | |
| pCMV | mNeonGreen-FKBP-CDK5RAP2 FL |
|  | mNeonGreen-CDK5RAP2 FL |
|  | mCherry-FRB-HSET |
|  | mCherry-HSET  mCherry-HSET(1-310)  mCherry-HSET(1-142) |
| pLAS3W | mStayGold-CDK5RAP2 |
| Bacteria surface display | |
| pDSG | Null  HSET FL  HSET (1-310)  HSET (1-142)  HSET (142-310)  HSET (311-673) |

**Supplemental Table 4| Primer sequences.**

|  | Forward sequence | Reverse sequence |
| --- | --- | --- |
| Primers for bacterial protein expression | | |
| EGFP-CDK5RAP2  (1-248) | ttaaagaggagaaatctcagatgtaatgacctgatgagaatgtgtcatctgg | ccagatgacacattctcatcaggtcattacatctgagatttctcctctttaa |
| EGFP-CDK5RAP2  (1-530) | GGGATCCCCGGAATTCATGGTGAGCAAGGGCGAGG | AGTCCATCATGAATTCCTTGTACAGCTCGTCCATGCC |
| EGFP-CDK5RAP2  (248-530) | CAGGACATGCGAATTCCTTGTACAGCTCGTCCATGCC | GATGCGGCCGCTCGAGTTAGAAGAGCACTTTTCTGTT |
| EGFP-CDK5RAP2  (531-749) | cggagtctaagatttcagattgataaggggcccacgcacc | ggtgcgtgggccccttatcaatctgaaatcttagactccg |
| EGFP-CDK5RAP2  (749-1100) | GGGGCCCCTGGGATCCTGTGATGGGGCCCACGCAC | GATGCGGCCGCTCGAGTCACTGATCAGTCCCCATCACAC |
| EGFP-CDK5RAP2  (531-1100) | GGGGCCCCTGGGATCCCAACAGCCACCAGGCAGC | GATGCGGCCGCTCGAGTCACTGATCAGTCCCCATCACAC |
| HSET (1-310) | TTCTGTTCCAGGGGCCCCTGGGATCCATGGATCCGCAGAGGTC | AGTCAGTCACGATGCGGCCGCTCGAGTCAGTTGCCCTTGAGTTCC |
| HSET(1-142) | TTCTGTTCCAGGGGCCCCTGGGATCCATGGATCCGCAGAGGTC | AGTCAGTCACGATGCGGCCGCTCGAGTCACTGACCCTTTAAGTCCCAG |
| Primers for transfection constructs | | |
| mNeonGreen-FKBP-CDK5RAP2 FL | TGGTGGTAGTGCTGGTGGTCCTCGAGCTATGATGGACTTGGTGTTGGAAGA | ATCAGTTATCTAGATCCGGTGGATCCTCAGGAGCCTGGTCTGCTGGGACT |
| mNeonGreen-CDK5RAP2 FL | CATGGACGAGCTGTACAAGCTCGAGATGGACTTGGTGTTGGAAGA | TCAGTTATCTAGATCCGGTGGATCCTCAGGAGCCTGGTCTGCTGGGA |
| mCherry-FRB-HSET | GCATGGACGAGCTGTACAAGGAATTCAGTGCTGGTGGTATCCTCTGGC | GAC CTC TGC GGA TCC ATG AAT TCA GCT CGA GGA CCA CCA GCA |
| mCherry-HSET | GACTCACTATAGGGAGACCCAAGCTTGCCACCATGGTGAGCAAGGGCGAGGA | TAGGGCCCTCTAGATGCATGCTCGAGTCACTTCCTGTTGGCCTGA |
| mCherry-HSET (1-310) | AAGGGCAACTGACTCGAGCATGCATCTAGAG | TGCTCGAGTCAGTTGCCCTTGAGTTCCTG |
| mCherry-HSET (1-142) | AAGGGTCAGTGACTCGAGCATGCATCTAGAG | TGCTCGAGTCACTGACCCTTTAAGTCCCAGG |
| mStayGold-CDK5RAP2 | AGACGTTAGAAGCTCATCTTGAATTCATGGACTTGGTGTTGGAAGAGG | GCCATTTGTCTCGAGGTCGAGAATTCTCAGGAGCCTGGTCTGCTG |
|  | Primers for bacterial surface display |  |
| Open pDSG primer | AGTTAATAAAGCGGCCGCTGCA | CCTAGTCGCACCATCAAAAAATATAACCGCA |
| pDSG-HSET FL |  | CAGCGGCCGCTTTATTAACTTCACTTCCTGTTGGCCTGAGCA |
| pDSG-HSET(1-142) | TATTTTTTGATGGTGCGACTAGGATGGATCCGCAGAGGTCC | CAGCGGCCGCTTTATTAACTTCACTGACCCTTTAAGTCCCA |
| pDSG-HSET(1-310) |  |  |
| pDSG-HSET(142-310) | TATTTTTTGATGGTGCGACTAGGTTATGTGACCTAAATGCAGAACTAAAAC | CAGCGGCCGCTTTATTAACTTCAGTTGCCCTTGAGTTCCTGCAG |
| pDSG-HSET(311-673) | TATTTTTTGATGGTGCGACTAGGATCCGTGTATTCTGCCGGGT | CAGCGGCCGCTTTATTAACTTCACTTCCTGTTGGCCTGAGCA |
| Primers for sequencing | | |
| HSET sequencing | GCTGCAGACATCAGAAGCAGCCCT | |
| mNeon-FKBP-sequencing | AGTGGCAAAAGGCCTTTACCGATGT | |
| GFP sequencing | ATGGTGAGCAAGGGCGAGGA | |
| CDK5RAP2 sequencing 1 | ATGGACTTGGTGTTGGAAGAG | |
| CDK5RAP2 sequencing 2 | CACAAATGGAGCATCAGAAGG | |
| CDK5RAP2 sequencing 3 | GCTCTTCTCAACAGCCACCA | |
| CDK5RAP2 sequencing 4 | GAGAAGGCCACATTATTACTGG | |
| CDK5RAP2 sequencing 5 | GCAAAGAAGCTACGAGATTGAC | |
| CDK5RAP2 sequencing 6 | CTGAATCAATGTGCTGAGCTG | |
| CDK5RAP2 sequencing 7 | CAAGCTATTGCAGTCTCTCCG | |
| CDK5RAP2 sequencing 8 | CTTCACTGTGAGCAGATTGG | |

**Supplemental Table 5|** **Detailed statistical values for box plots**

Fig. 1

|  | Fig. 1A | | Fig. 1B | | Fig. 1C | | Fig. 1D | |
| --- | --- | --- | --- | --- | --- | --- | --- | --- |
|  | I | M | I | M | I | M | I | M |
| Number of total values | 75 | 84 | 75 | 88 | 75 | 89 | 69 | 80 |
| Number of replicates | 3 | 3 | 3 | 3 | 3 | 3 | 3 | 3 |
| Minimum | 0.6950 | 1.640 | 0.4500 | 2.247 | 0.7320 | 1.848 | 0.8580 | 1.617 |
| 25% Percentile | 1.160 | 2.240 | 0.9400 | 2.868 | 1.230 | 2.952 | 1.700 | 2.527 |
| Median | 1.480 | 2.515 | 1.290 | 3.266 | 2.020 | 3.456 | 2.380 | 3.129 |
| 75% Percentile | 1.710 | 2.885 | 1.570 | 3.663 | 3.000 | 4.132 | 3.490 | 4.244 |
| Maximum | 2.210 | 3.580 | 2.640 | 4.277 | 7.240 | 5.088 | 6.790 | 5.698 |
|  |  |  |  |  |  |  |  |  |
| Mean | 1.444 | 2.567 | 1.284 | 3.260 | 2.388 | 3.526 | 2.759 | 3.395 |
| Std. Deviation | 0.4029 | 0.4313 | 0.4483 | 0.4972 | 1.525 | 0.7185 | 1.486 | 1.079 |

Fig. 2

|  | Fig. 2L | | | | Fig. 2M | | | |
| --- | --- | --- | --- | --- | --- | --- | --- | --- |
|  | DMSO | AZ82 | siControl | siHSET | DMSO | AZ82 | siControl | siHSET |
| Number of total values | 72 | 69 | 65 | 67 | 75 | 76 | 78 | 76 |
| Number of replicates | 3 | 3 | 3 | 3 | 3 | 3 | 3 | 3 |
| Minimum | 0.7110 | 0.9120 | 0.6210 | 0.5320 | 1.000 | 1.210 | 0.7630 | 0.9110 |
| 25% Percentile | 1.068 | 1.555 | 1.210 | 1.400 | 1.850 | 2.300 | 1.663 | 2.420 |
| Median | 1.805 | 2.310 | 1.850 | 2.710 | 2.430 | 3.310 | 2.110 | 3.260 |
| 75% Percentile | 2.835 | 3.665 | 2.680 | 5.170 | 3.500 | 4.793 | 2.910 | 5.255 |
| Maximum | 6.740 | 8.160 | 8.250 | 8.950 | 6.230 | 7.870 | 6.030 | 10.30 |
|  |  |  |  |  |  |  |  |  |
| Mean | 2.137 | 2.851 | 2.205 | 3.493 | 2.658 | 3.710 | 2.394 | 3.849 |
| Std. Deviation | 1.278 | 1.692 | 1.466 | 2.525 | 1.153 | 1.747 | 1.099 | 2.026 |

|  | Fig. 2N | | | | | | | |
| --- | --- | --- | --- | --- | --- | --- | --- | --- |
|  | Bipolar | | | | Multipolar | | | |
|  | DMSO | AZ82 | siControl | siHSET | DMSO | AZ82 | siControl | siHSET |
| Number of total values | 76 | 70 | 70 | 65 | 134 | 120 | 113 | 145 |
| Number of replicates | 3 | 3 | 3 | 3 | 3 | 3 | 3 | 3 |
| Minimum | 1.940 | 2.230 | 2.130 | 3.080 | 1.450 | 1.450 | 1.370 | 1.380 |
| 25% Percentile | 2.588 | 2.928 | 2.625 | 3.990 | 2.410 | 2.240 | 2.315 | 2.845 |
| Median | 3.035 | 3.505 | 2.955 | 4.810 | 2.940 | 2.715 | 3.030 | 3.580 |
| 75% Percentile | 3.480 | 4.040 | 3.360 | 6.440 | 3.620 | 3.420 | 3.595 | 5.600 |
| Maximum | 4.700 | 6.710 | 4.840 | 8.520 | 5.410 | 5.700 | 6.690 | 8.810 |
|  |  |  |  |  |  |  |  |  |
| Mean | 3.057 | 3.611 | 3.092 | 5.164 | 3.007 | 2.877 | 3.152 | 4.153 |
| Std. Deviation | 0.5931 | 0.9511 | 0.6467 | 1.499 | 0.8685 | 0.8941 | 1.174 | 1.889 |

|  | Fig. 2O | | | | | | | |
| --- | --- | --- | --- | --- | --- | --- | --- | --- |
|  | Bipolar | | | | Multipolar | | | |
|  | DMSO | AZ82 | siControl | siHSET | DMSO | AZ82 | siControl | siHSET |
| Number of total values | 60 | 62 | 66 | 60 | 117 | 132 | 126 | 119 |
| Number of replicates | 3 | 3 | 3 | 3 | 3 | 3 | 3 | 3 |
| Minimum | 1.770 | 2.130 | 1.770 | 2.130 | 1.590 | 1.460 | 1.380 | 1.360 |
| 25% Percentile | 2.258 | 2.668 | 2.455 | 2.735 | 2.130 | 2.298 | 1.888 | 2.430 |
| Median | 2.690 | 3.150 | 2.760 | 3.185 | 2.540 | 2.890 | 2.525 | 3.140 |
| 75% Percentile | 3.120 | 3.790 | 3.073 | 3.680 | 3.090 | 3.403 | 3.193 | 4.470 |
| Maximum | 4.420 | 6.750 | 4.360 | 8.700 | 4.630 | 4.710 | 4.550 | 8.510 |
|  |  |  |  |  |  |  |  |  |
| Mean | 2.694 | 3.305 | 2.801 | 3.409 | 2.679 | 2.865 | 2.614 | 3.556 |
| Std. Deviation | 0.5670 | 0.8775 | 0.5133 | 1.118 | 0.6853 | 0.7370 | 0.8085 | 1.585 |

Fig. 6

|  | Fig. 6D | | | Fig. 6E | | | Fig. 6F | | |
| --- | --- | --- | --- | --- | --- | --- | --- | --- | --- |
| HSET/Rapa | -/- | +/- | +/+ | -/- | +/- | +/+ | -/- | +/- | +/+ |
| Number of total values | 106 | 128 | 100 | 106 | 128 | 100 | 106 | 128 | 100 |
| Number of replicates | 3 | 3 | 3 | 3 | 3 | 3 | 3 | 3 | 3 |
| Minimum | 0.2434 | 0.4100 | 1.510 | 0.2434 | 7.700 | 8.500 | 0.002420 | 0.002384 | 0.004515 |
| 25% Percentile | 1.493 | 2.190 | 5.170 | 10.20 | 18.68 | 19.44 | 0.004361 | 0.006031 | 0.008350 |
| Median | 2.446 | 3.275 | 8.310 | 13.79 | 25.55 | 27.11 | 0.005306 | 0.008456 | 0.01071 |
| 75% Percentile | 3.999 | 4.863 | 13.01 | 17.22 | 34.00 | 32.21 | 0.006513 | 0.01174 | 0.01418 |
| Maximum | 8.798 | 12.59 | 23.87 | 41.88 | 74.37 | 70.55 | 0.01269 | 0.02819 | 0.02658 |
|  |  |  |  |  |  |  |  |  |  |
| Mean | 2.957 | 3.800 | 9.281 | 14.57 | 27.37 | 27.20 | 0.005854 | 0.009741 | 0.01147 |
| Std. Deviation | 1.943 | 2.370 | 5.388 | 7.679 | 12.67 | 10.18 | 0.002087 | 0.005328 | 0.004284 |

Extended Data Fig. 10

| Dynein inhibitor | Extended Fig. 10I | | Extended Fig. 10J | |
| --- | --- | --- | --- | --- |
| HSET/Rapa | - | + | - | + |
| Number of total values | 100 | 114 | 100 | 114 |
| Number of replicates | 3 | 3 | 3 | 3 |
| Minimum | 8.500 | 7.456 | 0.004515 | 0.003649 |
| 25% Percentile | 19.44 | 14.79 | 0.008350 | 0.006410 |
| Median | 27.11 | 20.93 | 0.01071 | 0.008191 |
| 75% Percentile | 32.21 | 33.72 | 0.01418 | 0.01323 |
| Maximum | 70.55 | 62.86 | 0.02658 | 0.02757 |
|  |  |  |  |  |
| Mean | 27.20 | 25.20 | 0.01147 | 0.01041 |
| Std. Deviation | 10.18 | 13.38 | 0.004284 | 0.005660 |

**References**

1 Tang, G., Cho, M. & Wang, X. OncoDB: an interactive online database for analysis of gene expression and viral infection in cancer. *Nucleic Acids Res* **50**, D1334-D1339, doi:10.1093/nar/gkab970 (2022).

2 Henkin, G., Chew, W. X., Nedelec, F. & Surrey, T. Cross-linker design determines microtubule network organization by opposing motors. *Proc Natl Acad Sci U S A* **119**, e2206398119, doi:10.1073/pnas.2206398119 (2022).

3 Chen, F. *et al.* Self-assembly of pericentriolar material in interphase cells lacking centrioles. *Elife* **11**, doi:10.7554/eLife.77892 (2022).
